# Gene Expression Changes in the Injured Spinal Cord Following Transplantation of Mesenchymal Stem Cells or Olfactory Ensheathing Cells

**DOI:** 10.1371/journal.pone.0076141

**Published:** 2013-10-11

**Authors:** Abel Torres-Espín, Joaquim Hernández, Xavier Navarro

**Affiliations:** Group of Neuroplasticity and Regeneration, Institute of Neurosciences, Department of Cell Biology, Physiology and Immunology, Universitat Autònoma de Barcelona, and Centro de Investigación Biomédica en Red sobre Enfermedades Neurodegenerativas (CIBERNED), Bellaterra, Spain; UMDNJ-Robert wood Johnson Medical School, United States of America

## Abstract

Transplantation of bone marrow derived mesenchymal stromal cells (MSC) or olfactory ensheathing cells (OEC) have demonstrated beneficial effects after spinal cord injury (SCI), providing tissue protection and improving the functional recovery. However, the changes induced by these cells after their transplantation into the injured spinal cord remain largely unknown. We analyzed the changes in the spinal cord transcriptome after a contusion injury and MSC or OEC transplantation. The cells were injected immediately or 7 days after the injury. The mRNA of the spinal cord injured segment was extracted and analyzed by microarray at 2 and 7 days after cell grafting. The gene profiles were analyzed by clustering and functional enrichment analysis based on the Gene Ontology database. We found that both MSC and OEC transplanted acutely after injury induce an early up-regulation of genes related to tissue protection and regeneration. In contrast, cells transplanted at 7 days after injury down-regulate genes related to tissue regeneration. The most important change after MSC or OEC transplant was a marked increase in expression of genes associated with foreign body response and adaptive immune response. These data suggest a regulatory effect of MSC and OEC transplantation after SCI regarding tissue repair processes, but a fast rejection response to the grafted cells. Our results provide an initial step to determine the mechanisms of action and to optimize cell therapy for SCI.

## Introduction

Spinal cord injury (SCI) leads to partial or complete loss of motor, sensory and autonomic functions and secondary impairments below the injury level, due to damage to the local circuitry of the spinal cord and the interruption of ascending and descending neural pathways. SCI results in a sequence of coordinated changes in gene and protein expression profile associated with physiopathological events, including hemorrhage, inflammatory and immune activation, excitotoxicity, oxidative stress, and neuronal activity imbalances [1], [2], [3], [4], [5].

Cell therapy has become a promising approach for repairing the injured spinal cord [6], [7], [8], [9], [10]. Several pre-clinical studies have demonstrated that transplantation of mesenchymal stromal cells (MSC) [11], [12], [13], [14], [15] or olfactory ensheathing cells (OEC) [16], [17], [18], [19], [20], [21] reduces tissue damage and improves functional outcomes in different models of SCI, although other studies failed to replicate such beneficial results [22], [23], [24], [25], [26]. Little is known about the mechanisms underlying the potential benefits after cell grafting into the injured spinal cord. Regarding the MSC it has been suggested that the effects are due to their capability to secrete and/or induce the expression of protective molecules such as BDNF and GDNF [12], [15], to modulate inflammation [27], [28] and to generate a more permissive environment for axonal regeneration and neural tissue reconstruction [12], [13], [29], [30]. The beneficial actions of OEC include the ability of these cells to modulate and interact with reactive astrocytes [31], [32], to induce neoangiogenesis [33], [34], to remyelinate naked axons [35], to modulate the immune response [31], [33], [34] and to promote axonal regeneration [17], [18], [36], [37].

Although a number of studies have investigated the changes in gene expression profile after different types of SCI in laboratory animals, no studies have focused on the analysis of gene expression changes triggered by transplanted cells in the lesioned spinal cord. Such information may be of importance to better understand the cellular and molecular mechanisms modulated by the transplanted cells. In the present work, we analyzed for the first time the gene expression profiles of the spinal cord that received an acute or 7 days delayed graft of MSC or OEC following a contusion injury. Our results confirm that SCI causes several changes in gene transcription, and the injection of cells significantly modifies some of the pathways affected after injury. Transplantation of both MSC and OEC leads to over expression of genes involved in tissue repair during the acute phase of the injury, and the decline during subacute time. Our results further indicate how these cells contribute to regulating the wound repair response after SCI, and could explain the beneficial effects provided by the transplantation. On the other hand, a large number of genes associated with the immune response were also found up-regulated, indicative of cell rejection.

## Materials and Methods

### Primary Cell Cultures

Primary cultures of MSC and OEC were set up from P22 male Sprague-Dawley rats. The animals were euthanized with CO_2_.

#### MSC culture and characterization

Tibias and femurs were placed on cool phosphate buffered saline (PBS) and the epiphyses were removed. The diaphyses of the bones were flushed with PBS using a syringe and the marrow was homogenized. The extract was filtered through a 70 µm nylon mesh and recovered by centrifugation for 10 min at 1500 rpm. The pellet was resuspended in growth medium: α-MEM with L-glutamine (Life Technologies, Grand Island, NY, USA) supplemented with 20% heat-inactivated fetal bovine serum (FBS) (Lonza, Verviers, Belgium), 2 mM L-glutamine (Life Technologies) and 100 units/ml penicillin-streptomycin (Life Technologies, 100x); then plated in 100 mm culture dishes (Iwaki, Asahi Technoglass, Chiba, Japan) at a density of 5·10^6^ cells/cm^2^. After 24 h, the supernatant containing non-adherent cells was removed and fresh medium was added. When the culture was near confluence, every 4–5 days, the cells were detached using PBS with 0.05% trypsin (Life Technologies) and 0.04% EDTA (Sigma, St. Louis, MO) and re-plated at 5,000 cells/cm^2^. Cells were passaged 3–4 times and expanded to 80–90% of confluence. The cultured MSC were characterized by their expression of CD90 and CD29 (about 98% of the cells in the culture expressed these markers) but not of CD11b and CD45 surface markers (expressed by less than 1% of the cells), and by their differentiation capability to adipocytes and osteoblasts using methods previously described [38]. For analysis of adipogenesis, cells were fixed for 20 min with 4% paraformaldehyde in phosphate buffer (PB); the adipocytes were labeled using 60% Oil red O stock solution (0.5% Oil red O in isopropanol, Sigma) for 15 min and washed with distilled water. For osteocytes labeling, cells were fixed using 70% ethanol pre-cooled for 1 h at 4°C, washed and incubated during 30 min with 0.1 mg/ml Alizarin red solution (Sigma) in distilled water.

#### OEC culture and characterization

Olfactory bulbs were aseptically removed and stored in cold Hank’s balanced salt solution (HBBS) with calcium and magnesium. The meningeal layer was stripped off with fine forceps, and the tissue was enzymatically (trypsin 0.25%, collagenase A 1 mg/ml, and DNAse I 1 mg/ml) and mechanically dissociated. The cells were recovered by centrifugation in Dulbecco’s minimum essential medium nutrient mixture F-12 Ham (DMEM) and seeded onto 25 cm^2^ flasks coated with poly-L-lysine and incubated in 5% CO_2_ at 37°C. Culture medium was DMEM supplemented with 10% FBS (Life Technologies). Cells were kept in culture for at least 7 days. For purification, the cells were incubated with mouse anti-p75NGFR antibody (1∶100, Chemicon, MAB365), then immunopurified with goat anti-mouse IgG microbeads (Miltenyi Biotec) using MACS separation (Miltenyi Biotec). The OEC culture purity was at least 75%. For immunocytochemistry OEC cultures were fixed with 4% paraformaldehyde in PBS for 30 minutes, then incubated for 24 h with primary antibodies anti-p75NGFR and anti-S100 (1∶200, DiaSorin), and after washing incubated 1 h with secondary antibodies Cy3-conjugated donkey anti-mouse (1∶200, Jackson Immunoresearch) or Alexa 488 donkey anti-mouse (1∶200, Invitrogen).

### Cell Labeling

For identification after grafting, both cell types were transfected with a lentiviral vector encoding for green fluorescent protein (GFP) under EF1α promoter. MSC in passage 2 were plated at 2000 cells/cm^2^ and incubated with lentiviruses at MOI of 10 during 48 h. Then, the medium was changed and the cells cultured as described above. OEC transduction was done at 2–3 days of selection using the same lentiviruses at MOI of 50 for 24 hours. Then, the medium was replaced by complete culture medium and the cells cultured for 5 more days. Around 96% of transfection efficacy was determined by GFP+ cell counting for both MSC and OEC.

### Spinal Cord Injury and Cell Transplantation

Adult Sprague-Dawley female rats (9 weeks old; 250–300 g) were used. The animals were housed with free access to food and water at room temperature of 22±2°C. The experimental procedures were approved by the ethical committee of the Universitat Autònoma de Barcelona (Procedure Number: 1169) in accordance with the European Directive 86/609/EEC.

Under anesthesia with ketamine (90 mg/kg) and xylacine (10 mg/kg) and aseptic conditions, a longitudinal dorsal incision was made to expose T6–T10 spinous processes. After laminectomy of T8–T9 vertebra, the spinal cord was subjected to a contusion of 200 Kdyns using the Infinite Horizon Impactor (Precision System and Instrumentation, Kentucky, USA). The animals for the transcriptome study were divided in 6 groups. Three groups of rats were transplanted acutely, 30 min after operation (0 dpo), with vehicle (VE 0, n = 8), with MSC (MSC 0, n = 8) or with OEC (OEC 0, n = 8). Other three groups of rats were transplanted at 7 days post-lesion with vehicle (VE 7, n = 8), MSC (MSC 7, n = 8) or OEC (OEC 7, n = 8). For assessment of cell localization after grafting, GFP+ cells (gMSC or gOEC) were transplanted either acutely or 7 days post-lesion in the corresponding groups gMSC 0 (n = 3), gOEC 0 (n = 3), gMSC 7 (n = 3) and gOEC 7 (n = 3). For transplantation, cells were suspended in L15 medium (Life Technologies) at 50,000 cells/µl and maintained in ice during the time of surgery. Using a glass needle (100 µm internal diameter, Eppendorf, Hamburg, Germany) coupled to a 10 µl Hamilton syringe (Hamilton #701, Hamilton Co, Reno, NV, USA), 3 µl of the corresponding cell suspension or vehicle (L15) were intraspinally injected at the epicenter and at 2 mm rostrally and caudally, for a total of 450,000 cells per rat. Injections were made at a perfusion speed of 2 µl/min controlled by an automatic injector (KDS 310 Plus, Kd Scientific, Holliston, MA, USA), and the tip of the needle was maintained inside the cord tissue 3 min after each injection to avoid liquid reflux. The wound was sutured and the animals allowed to recover in a warm environment. Bladders were expressed twice a day until reflex voiding of the bladder was re-established. To prevent infection, amoxicillin (500 mg/l) was given in the drinking water for one week.

### Sample Preparation

The rats for the transcriptome study in each experimental group were sacrificed randomly at 2 (n = 4) or 7 (n = 4) days post cell injection (dpi). The groups were identified according to the code *transplant day.day_of sacrifice_post-treatment*. For example, the group of animals transplanted with MSC at 7 days post-lesion and sacrificed 2 days after treatment was labeled as MSC 7.2. Animals were decapitated after deep anesthesia, and a spinal cord segment (5 mm long) centered in the contusion epicenter was harvested and maintained in RNA-later solution (Qiagen, Barcelona, Spain). A spinal cord segment of intact animals (n = 4) was also obtained at the same vertebral level as for the injured animals. The samples were processed for mRNA analysis following the manufacturer instructions. The total RNA of each sample was extracted with RNeasy mini kit (Qiagen), including a DNase step (RNase free DNase set, Qiagen).

For cell tracking, animals injected with gMSC or gOEC were sacrificed at 7 days post-injection. The rats were deeply anesthetized (pentobarbital 60 mg/kg i.p.) and intracardially perfused with 4% paraformaldehyde in PBS. The spinal cord segment from 1 cm rostral to 1 cm caudal of the injury epicenter (2 cm total length) was harvested and post-fixed in the same fixative solution for 24 h and cryopreserved in 30% sucrose. Longitudinal spinal cord sections 30 µm thick were cut with a cryotome (Leica CM190, Leica Microsystems, Wetzlar, Germany) and distributed in 12 series of 8 sections (separated by 360 µm) each.

### Immunohistochemistry

Spinal cord sections of GFP+ cells transplanted rats were processed for immunohistochemistry against GFP. Tissue sections were blocked with TBS-0.3% Triton-5% fetal bovine serum and incubated for 24 h at 4°C with the antibody rabbit anti-GFP (1/200, Life Technologies). After washes, sections were incubated for 2 h at room temperature with secondary antibody donkey anti-rabbit AlexaFluor 488 (1/200, Jackson Immunoresearch). Slides were dehydrated and mounted with Citoseal 60 (Thermo Fisher Scientific, Madrid, Spain). Images were obtained with a digital camera (Olympus DP50) attached to the microscope (Olympus BX51). Analysis of the area occupied by GFP+ cells was performed using 8 spinal cord sections (separated by 360 µm between pairs) of each animal. Images of the spinal cord injured segment were taken at 40x with the same setting and the total section was mounted using Photoshop software (Adobe Systems Inc.). The microphotographs were analyzed using ImageJ software. The GFP labeled area in each section was measured after defining a threshold for background correction. The volume of the graft, spared tissue, cavity and total spinal cord injured segment were calculated using the Cavalieri’s estimator of morphometric volume. The statistical analysis was performed using one-way ANOVA.

### Microarray

The microarray hybridization and the statistical processing of raw data were performed by a specialized service (Scientific and Technical Support Unit and Statistics and Bioinformatics Unit, Vall d’Hebron Research Institute, Barcelona, Spain). For the gene expression an Affymetrix RAT Exon/Gene 1.1 ST chip array was used according to the manufacturer protocol, analyzing each animal individually as biological replicates of four animals per experimental condition. The array data are available in Gene Expression Omnibus database (GEO, National Centre of Biotechnology Information), accession number GSE46988.

#### Data analysis

The images of hybridized microarrays were processed with the Expression Console software (Affymetrix). Raw expression values obtained directly from.CEL files were pre-processed using the RMA method [39], a three-step process that integrates background correction, normalization and filtering of probes values. Data were first submitted to non-specific filtering to remove low signal genes (those genes whose mean signal in each group did not exceed a minimum threshold) and low variability genes (those genes whose standard deviation between all samples did not exceed a minimum threshold). The selection of differentially expressed genes between conditions was based on a linear model analysis with empirical Bayes moderation of the variance estimated following the methodology developed by Smyth [40] and implemented in the limma Bioconductor package. To determine the main effects of the injury, the gene expression profile of each injured group was compared to non injured animals and cut-off *P* value <0.05 and fold change (FC) >1.5 were applied to select the differentially expressed genes. To determine the gene expression changes after cell transplantation, each gene expression profile of MSC or OEC groups was compared to the correspondent vehicle group. In this case, the cut-off for differentially expressed genes were a *P* value <0.05 and a FC >1.4.

All the statistical analyses were done using the free statistical language R and the libraries developed for microarray data analysis by the Bioconductor Project (www.bioconductor.org).

### Microarray Validation

To validate the results obtained by microarray analysis, *in silico* comparison of the SCI differential expressed genes lists and RT-PCR of target genes were performed.

#### *In silico* validation

Three different data profiles were selected in the GEO database: GSE464 (spinal cord injury contusion and regeneration time course in rats: above T9 (RG-U34A)), GSE5296 (spinal cord injury contusion model in mice: time course), and GSE22161 (comparative gene expression analysis of thoracic spinal cord from G93A SOD1 mutant rats and from wild type littermates following mild compression injury). For the comparison of the GEO profile with our results, up-regulated and down-regulated lists of genes were obtained from the GEO data analysis tools, filtered with our microarray profile to eliminate the probes not assessed in our chip, and compared with the corresponding up-regulated and down-regulated lists that we obtained from our samples. The percentages of concordant and discordant genes with respect to the total changed genes in the GEO data up-regulated and down-regulated profiles were calculated. Thus, if one gene appeared up-regulated at the same time in the GSE464 and in our results this gene contributed to the percentage of concordance. Genes that were not coincident in the compared list contributed to the percentage of discordance, in which we distinguished the genes that changed in the GEO profile but not in our data and the genes that changed in opposite direction than in our results.

#### RT-PCR

1 µg RNA of each sample was reverse-transcribed using 10 µmol/L DTT, 200 U M-MuLV reverse transcriptase (New England BioLabs, Barcelona, Spain), 10 U RNase Out Ribonuclease Inhibitor (Invitrogen), 1 µmol/L oligo(dT) and 1 µmol/L of random hexamers (BioLabs, Beverly, MA, USA). The reverse transcription cycle conditions were 25°C for 10 min, 42°C for 1 h and 72°C for 10 min. We analyzed the mRNA expression by means of specific primer sets (Table S1). Glyceraldehyde 3-phosphate dehydrogenase (GADPH) expression was used to normalize the expression levels of the different genes of interest. Gene-specific mRNA analysis was performed by SYBR-green PCR using the MyiQ5 PCR detection system (Bio-Rad Laboratories, Barcelona, Spain). We previously fixed the optimal concentration of the cDNA to be used as template for each gene analysis to obtain reliable CT (threshold cycle) values for quantification. Four samples were used per condition and each one was run in duplicate. The thermal cycling conditions comprised 3 min polymerase activation at 95°C, 40 cycles of 10 s at 95°C for denaturation and 30 s at 62°C for annealing and extension, followed by a DNA melting curve for determination of amplification specificity. CT values were obtained and analyzed using BioRad Software. Fold change in gene expression was estimated using the CT comparative method (2^−ΔΔCT^) normalizing to GADPH CT values and relative between pairs of samples.

### Cluster Analyses

Hierarchical cluster analyses were performed using the algorithms for complete linkage and correlation (uncentered) similarity metrics in the software Cluster 3.0 and visualized using TreeView, both developed by Eisen et al. (1998) [41]. To determine the similarity of the experimental conditions, an array hierarchical clustering of the transplanted groups (MSC 0.2, MSC 0.7, MSC 7.2, MSC 7.7, OEC 0.2, OEC 0.7, OEC 7.2, OEC 7.7) was performed. To compare the differentially expressed profiles between MSC (MSC vs. VHC) and OEC (OEC vs. VHC), a gene hierarchical clustering was performed for each experimental condition. In this analysis a specific list of genes were selected from the resulted clusters using the criteria: genes exclusively up- or down-regulated in MSC or OEC transplanted animals and genes that were up- or down-regulated in both cell injected group.

### Analysis of Biological Meaning

To investigate the biological meaning, term enrichment analysis in the Gene Ontology (GO, http://www.geneontology.org/) and functional annotation GO term clustering analysis were performed [42] for each list of genes selected in the hierarchical clustering. The Database for Annotation, Visualization and Integrated Discovery (DAVID, v6.7) (National Institute of Allergy and Infectious Diseases (NIAID); http://david.abcc.ncifcrf.gov/home.jsp) was used for DAVID’s GO biological process (GOTERM_BP_ALL). The GO terms were classified by functional annotation clustering analysis, where the list of selected genes (the sample) were compared to a reference set (the whole probes in the Affymetrix chip used) with the following options: similarity term overlap = 3; similarity threshold = 0.5; initial group membership = 2; final group membership = 2; multiple linkage threshold = 0.05; EASE = 0.05. The functional annotation clusters were ranked from largest to smallest enrichment score (ES) and the GO terms associated to every cluster were ranked from smallest to largest *P* value. Moreover, every cluster was labeled with a representative name of the GO terms included in the cluster.

### Data Presentation

A summary of the array analysis is presented including heat maps of array and gene hierarchical clustering, a summary of GO functional clusters for each selected cluster and the number of up- and down-regulated genes. The whole information of the functional annotation cluster classification, with the GO terms included in each cluster, the number of genes in every enriched GO term (G) and the p value of the clustering, is also presented in Tables S2–S20.

## Results

### Cell Localization in the Injured Spinal Cord

**Figure 1:**
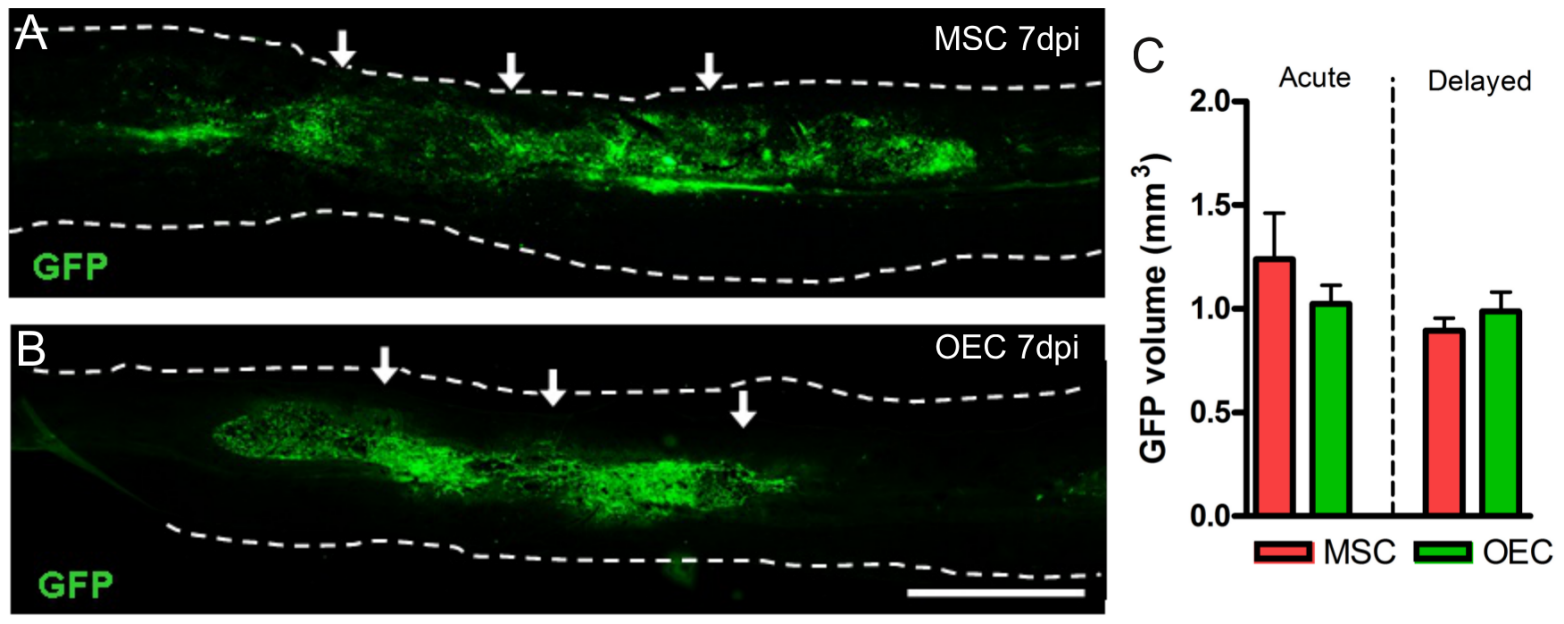
Grafted cell survival in the injured spinal cord. GFP labeled MSC and OEC were transplanted into the spinal cord after contusion at acute or delayed time. At 7 days post injection (dpi), both OEC (A) and MSC (B) were localized in the spinal cord parenchyma (delimited by dotted lines), surrounding the injection points (arrows). The volume quantification of the GFP signal showed no significant differences between groups (C). Scale bar = 2000 µm.

In order to assess the presence of the grafted cells after transplantation we labeled MSC and OEC with GFP. The GFP+ cells were localized into the injured spinal cord 7 days after acute and delayed transplantation (Fig. 1A,B). The quantification of the GFP signal volume revealed no statistical differences between any groups of cell transplantation (Fig. 1C).

### Microarray Validation

Microarray data were validated by both *in silico* and RT-PCR quantitative analyses. For the *in silico* comparison, the transcriptional profile of the SCI samples was contrasted with three microarrays data published in the GEO database: GSE464, GSE5296 and GSE22161. After filtering genes that were differentially expressed, we found that the genes that were up-regulated and down-regulated in our experiments were very similar to those found in a dataset obtained from rat spinal cord following a mild contusion injury (GSE464 profile) (Fig. S1A). Indeed, coincidence of genes that were up-regulated and down-regulated was 88.5% and 79.1%, respectively, at day 2, and 90.8% and 75.3%, respectively, at day 7 post-injury. Our results were also analogous to those obtained after mild spinal cord contusion in mice (GSE5296 profile) since the matching of genes that were up-regulated and down-regulated at day 7 post-injury was 79.4% and 84.1%, respectively. On the other hand, less than 50% of the genes (37.6% and 45% for up- and down-regulated genes, respectively) coincided with the gene expression profile obtained 7 days after spinal cord compression injury (GSE22161 profile). Therefore, these comparisons indicate that transcriptome changes that occur in the spinal cord are largely dependent on the type of primary lesion.

To validate the gene expression changes observed in the rat spinal cord after contusion, we analyzed by RT-PCR 9 targeted genes identified in the microarray (see Table S1). The expression changes of these 9 genes were compared between the microarray and the RT-PCR results for each experimental group included in the study (Fig. S1B). The results showed a significant correlation comparing both methods (Pearson’s r = 0.87, p<0.0001), evidencing the reliability of our microarray data.

### Biological Changes after Acute Cell Transplantation

**Figure 2:**
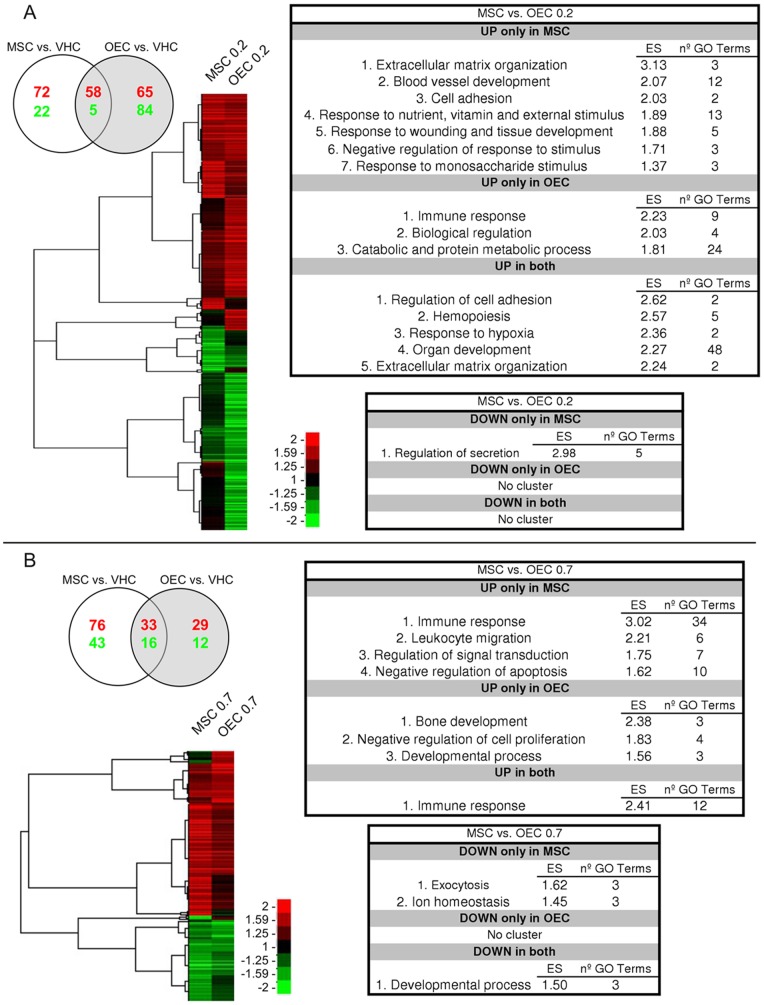
Changes in gene expression after acute MSC and OEC transplantation. The figure shows the gene profiles comparison between MSC and OEC at 2 (A) and 7 (B) days after acute transplantation in rats with a spinal cord contusion. Each panel contains the Venn diagram (top left) showing the number of differentially expressed genes (in red, up-regulated genes; in green, down-regulated genes), the heat map of the corresponding hierarchical clustering with the clusters trees, and the summary tables of the functional annotation analysis results. In the heat map the columns represent the mean of four animals analyzed per group.

Acute cell transplantation induced changes in the transcriptome profile of the injured spinal cord. At 2 days after treatment 306 genes were found changed in comparison with vehicle injection. Of them, 72 genes were up- and 22 down-regulated exclusively by the MSC graft, 65 genes were up- and 84 genes down-regulated exclusively by the OEC graft, whereas 58 and 5 genes were observed up- and down-regulated, respectively, in both MSC and OEC groups (Fig. 2A). The functional annotation clusters associated with the different genes are summarized in Figure 2A (full information in Tables S3–S10).

At 7 days after acute transplantation we found a lower number of genes that changed their expression in comparison to results at 2 days (209 vs. 306). The MSC graft induced exclusive up-regulation of 76 and down-regulation of 43 genes. The OEC graft induced exclusive up-regulation of 29 and down-regulation of 12 genes. Both cell grafts were associated with 33 up-regulated genes and 16 down-regulated genes (Fig. 2B). The functional annotation clusters associated with the different genes are summarized in Figure 2B (full information in Table S11–S20).

### Biological Changes after Delayed Cell Transplantation

**Figure 3:**
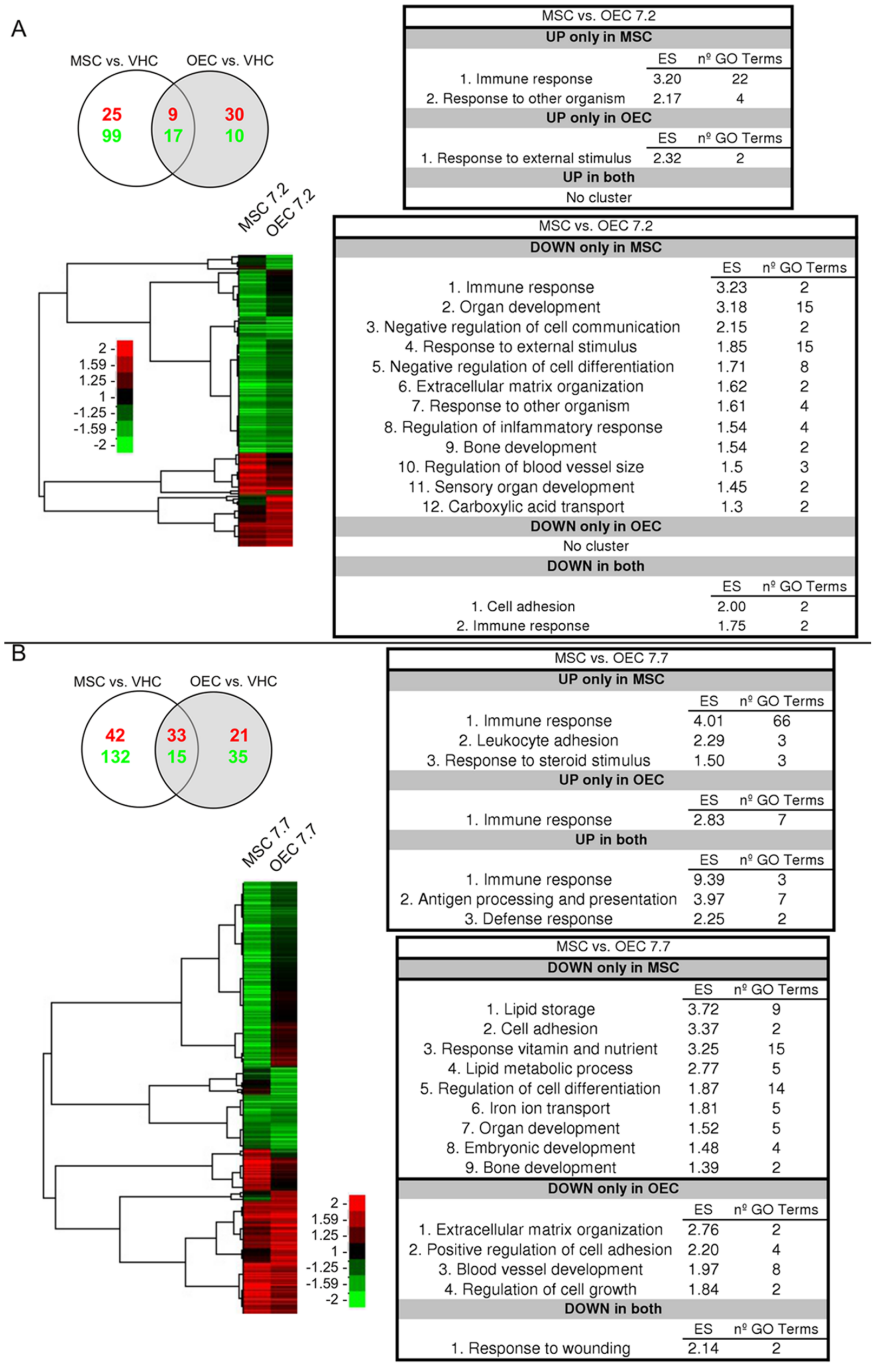
Changes in gene expression after delayed MSC and OEC transplantation. The figure shows the gene profiles comparison between MSC and OEC at 2 (A) and 7 (B) days after one week delayed transplantation in rats with a spinal cord contusion. Each panel contains the Venn diagram (top left) showing the number of differentially expressed genes (in red, up-regulated genes; in green, down-regulated genes), the heat map of the corresponding hierarchical clustering with the clusters trees, and the summary tables of the functional annotation analysis results. In the heat map the columns represent the mean of four animals analyzed per group.

At 2 days after delayed cell transplantation, 190 genes changed their expression by a cell graft compared with vehicle injection. 25 of these genes were up- and 99 were down-regulated exclusively by MSC, while 30 genes were up- and 10 down-regulated only by OEC. Both treatments up-regulated the same 9 genes and down-regulated the same 17 genes (Fig. 3A).

At 7 days after delayed cell transplantation the expression of 308 genes was changed in comparison with vehicle injection. 42 of these genes were up- and 132 were down-regulated exclusively by MSC, and 21 genes were up- and 35 genes were down-regulated exclusively by OEC. Both cell transplants up-regulated the same 33 genes and down-regulated other 15 genes (Fig. 3B). The list of functional annotation clusters associated with genes up- or down-regulated by each cell transplant or by both are detailed in Figure 3A and B.

### Shared Gene Expression Changes by Cell Transplantation

**Figure 4:**
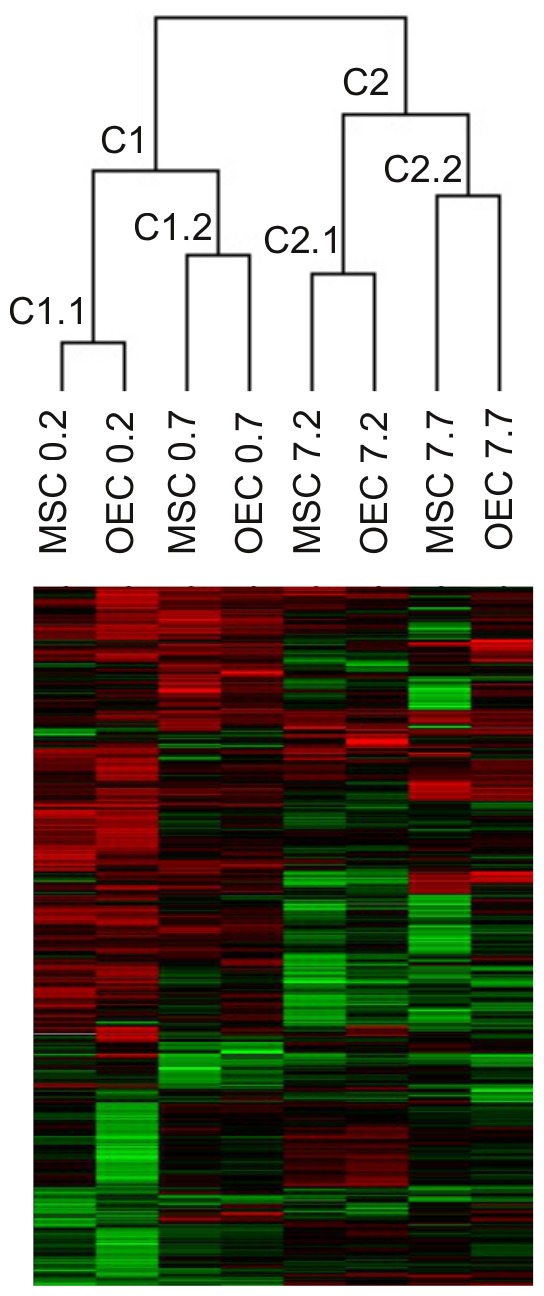
Hierarchical clustering of the array. The experimental conditions were submitted to hierarchical clustering and the corresponding heat map and cluster tree are shown. The clustering analysis determines a classification in two big clusters including the acute (C1) and delayed transplant (C2). In these two clusters the MSC and the OEC induced profile at 2 days (C1.1 and C2.1) and at 7 days (C1.2 and C2.2) after acute or delayed treatment were grouped.

The hierarchical clustering of the arrays performed between the 8 cell therapy experimental conditions revealed two profile similarities (Fig. 4). The first level of clustering grouped the 4 groups of acute transplant in one cluster and the delayed transplant groups in another cluster (C1 and C2 in Fig. 4). Within these two clusters, the profiles found at early time (2 days) after injection of MSC and OEC were clustered together (C1.1 and C2.1 in Fig. 4), and the profiles of later time (7 days) after injection of both types of cells were in the same cluster (C1.2 and C2.2 in Fig. 4). These results indicate that the pattern of gene changes induced by cell transplant is more similar between MSC and OEC than the temporal changes triggered by the same transplanted cells. The shared processes are mainly related to tissue repair, such as extracellular matrix (ECM) organization, organ development, response to wounding and cell adhesion. In addition, the immune response events appear modulated by both cells.

### ECM Organization after Cell Transplantation

**Figure 5:**
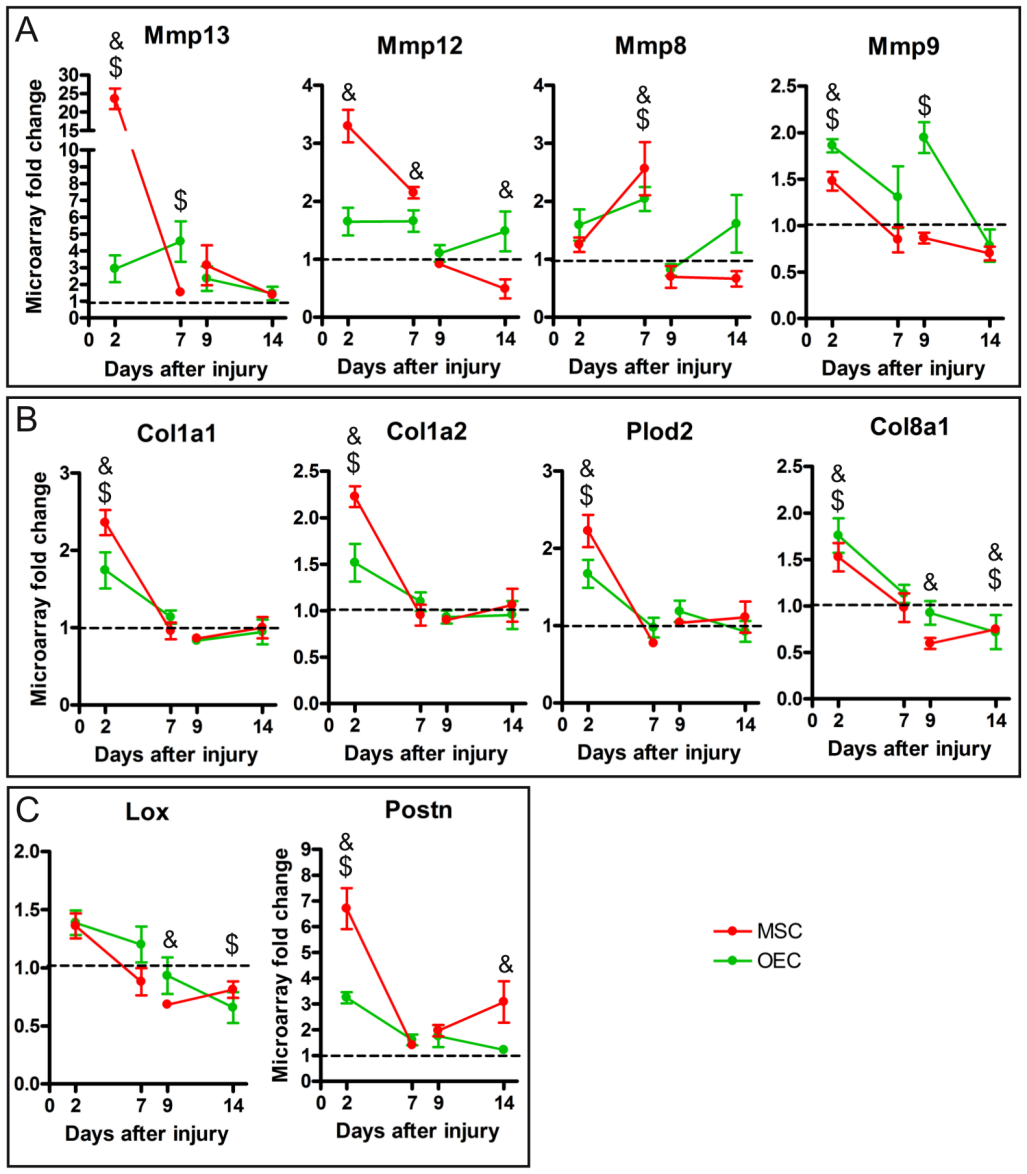
ECM related genes. Graphical representation of genes related to ECM remodeling, such as matrix metalloproteases (A), collagen associated genes (B) and other genes related to collagen interaction proteins (C). Each graph shows the fold changes of the corresponding gene after acute (2 and 7 days post injury) and delayed (9 and 14 days post injury) cell transplantation in comparison to vehicle values. Data are represented as mean ± SEM. $ p<0.05 OEC vs. vehicle, & p<0.05 MSC vs. vehicle.

ECM modulation is one of the processes shared by MSC and OEC transplants that include some of the most changed genes in the array, suggesting that ECM remodeling is an important mechanism modified by cell transplantation. Figure 5 shows some of the genes related to ECM organization that most changed their expression. These changes involved an early up-regulation of the matrix metalloproteases Mmp13, Mmp12, Mmp9 and Mmp8 (Fig. 5A), pro-collagen type I Col1a1 and Col1a2, Plod2, other member of the collagen family, such as Col8a1 (Fig. 5B), and genes involved in ECM formation, Lox (lysyl oxidase), the enzyme responsible to link collagen with elastin, and Postn (periostin) (Fig. 5C). While some genes showed similar temporal expression between MSC and OEC transplants, including Mmp13, Mmp8, Col1a1, Col1a2 and Plod2, other genes had a different temporal pattern. The Mmp12 expression was increased only by MSC acute transplantation. Other differences between cell grafts were observed in Mmp9 and Postn expression after the delayed treatment. All together these observations indicate an important alteration of the ECM remodeling process after transplantation of both types of cells.

### Immune Response Against the Grafted Cells

**Figure 6:**
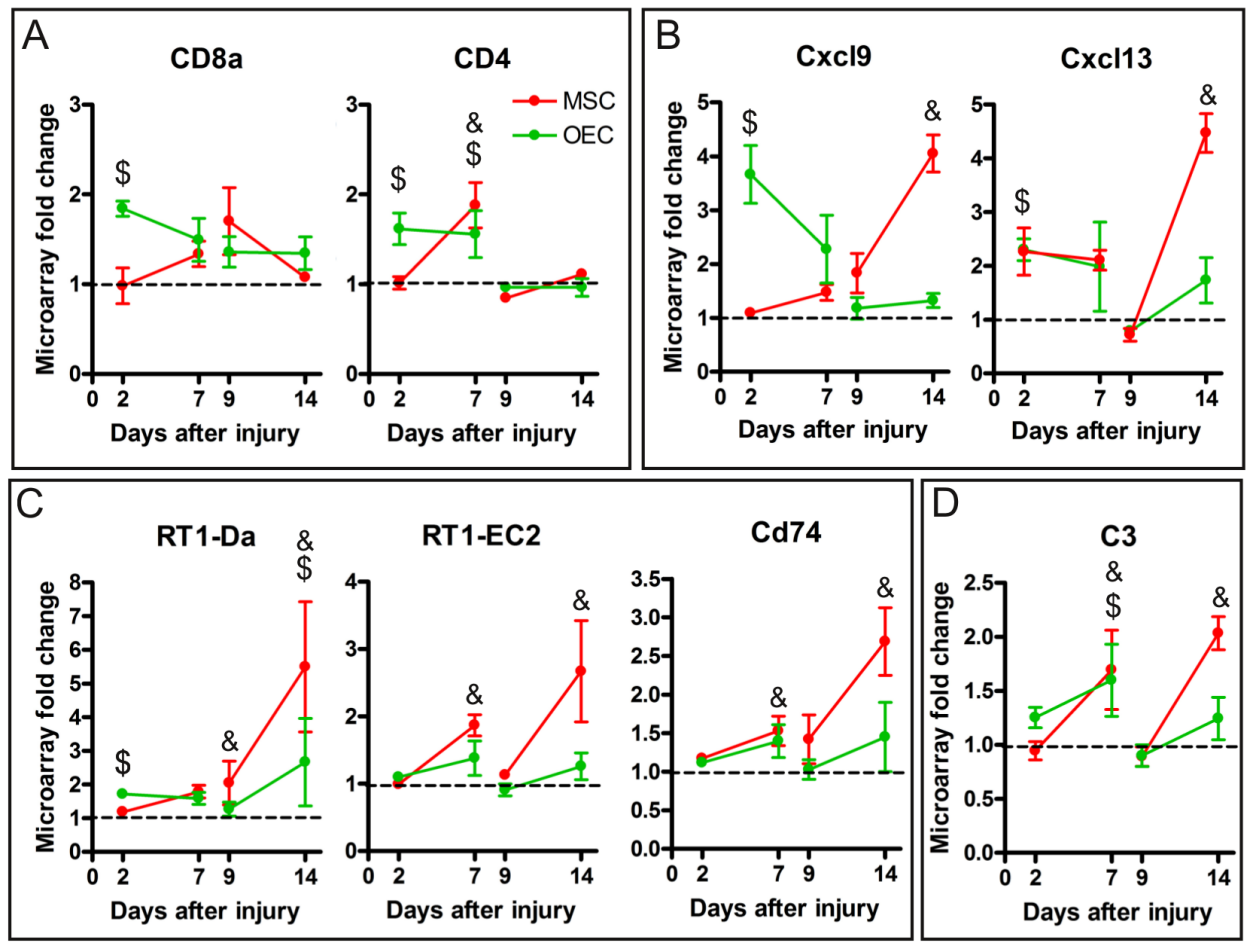
Rejection response related genes. Graphical representation of some genes related to immune response, such as lymphocyte markers (A), cytokines (B), major histocompatibility complex I and II (C) and C3, the complement component 3 (D). Each graph shows the fold changes of the corresponding gene after acute (2 and 7 days post injury) and delayed (9 and 14 days post injury) cell transplantation in comparison to vehicle value. Data are represented as mean ± SEM. $ p<0.05 OEC vs. vehicle, & p<0.05 MSC vs. vehicle.

Most of the GO terms clustered in the immune response functional group were related to leukocyte migration, lymphocyte activation, response to other organism, adaptive immune response and antigen presentation (Tables S6, S11, S15, S16, S17). Associated with these processes we found significant up-regulation of CD8a and CD4 after acute OEC transplant, but only of CD4 after acute MSC transplant (Fig. 6A). A differential pattern of expression over time was also observed for the Cxcl9 gene (Fig. 6B), that was up-regulated after an acute OEC graft and after a delayed MSC graft. Both acute transplants induced up-regulation of Cxcl13, but only the delayed transplant of MSC induced its overexpression. RT1-Da, RT1-EC2, CD74 (Fig. 6C) and C3 (Fig. 6D) showed similar temporal expression patterns, although with some significant differences between cell grafts. In general, the four genes increased expression from 2 to 7 days after cell grafting. However, a stronger up-regulation of the four genes was observed by delayed grafting of MSC than of OEC. These data probably reflect an immune response triggered against the grafted cells.

### Specific Changes Induced by Transplanted Cells

**Figure 7:**
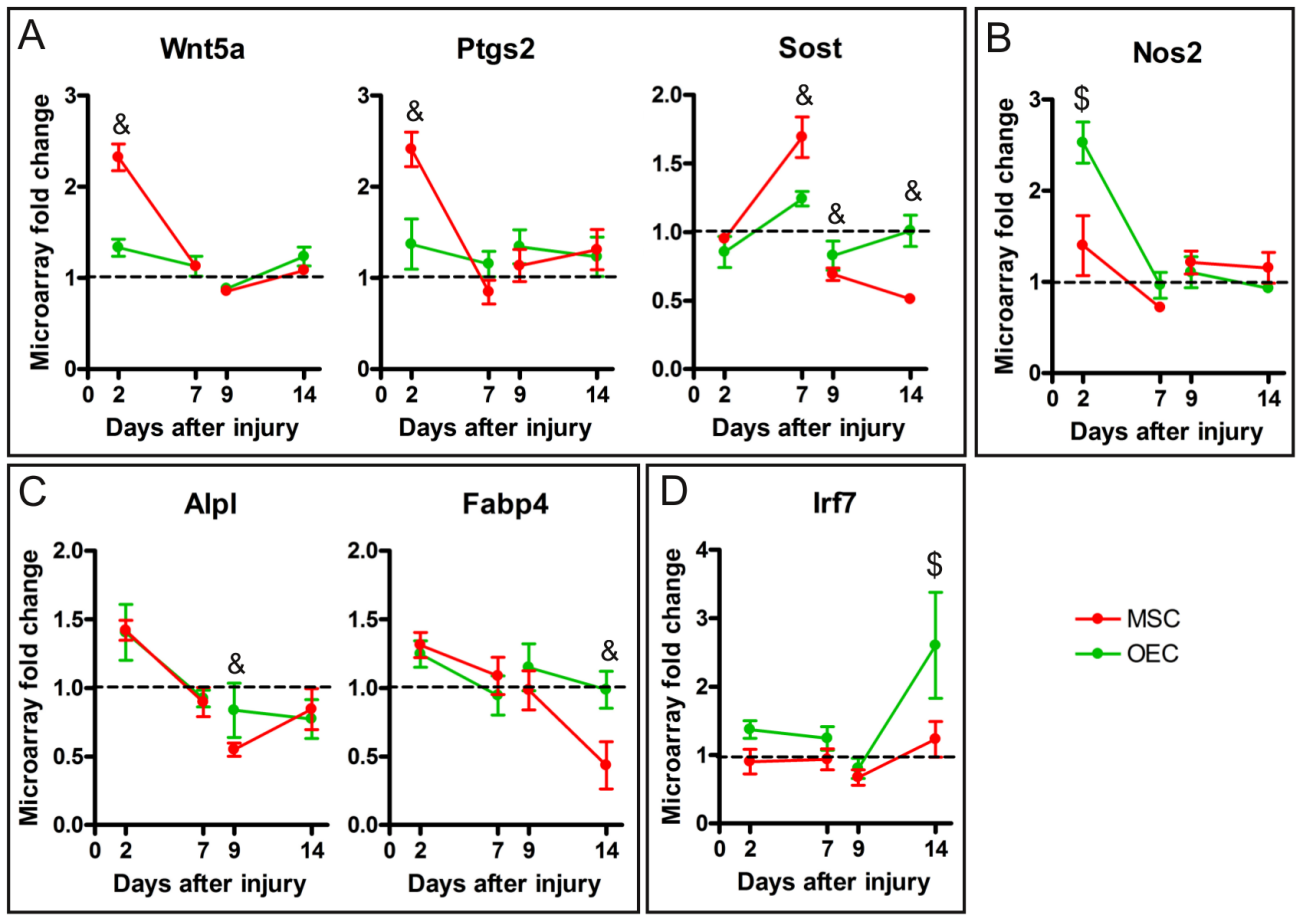
Specific genes modulated by cells. Graphical representation of some genes that were specifically expressed by acute MSC (A), acute OEC (B), delayed MSC (C) or delayed OEC (D) transplantations. Each graph shows the fold changes of the corresponding gene after acute (2 and 7 days post injury) and delayed (9 and 14 days post injury) cell transplantation in comparison to vehicle value. Data are represented as mean ± SEM. $ p<0.05 OEC vs. vehicle, & p<0.05 MSC vs. vehicle.

Besides the numerous similarities between gene profiles found after both MSC and OEC grafts in the SCI, each type of cells specifically modulated other biological events. Thus, during the acute phase of SCI the presence of MSC induced an early enhancement of genes related to response to nutrient, vitamins and external stimulus (Fig. 2A). Within these processes Wnt5a and Ptgs2 were the most up-regulated genes (Fig. 7A). At 7 days after acute MSC transplant Wnt5a and Ptgs2 returned to normal expression and the negative regulation of apoptosis increased (Fig. 2B). The most important gene associated to this process was Sost (Sclerostin) (Fig. 7A). In the case of OEC transplants, a large increase of catabolic and protein metabolic processes was observed early after acute injection (Fig. 2A), with a particular up-regulation of Nos2 gene (Fig. 7B). At 7 days after acute OEC transplant, genes modulated by OEC were related to tissue repair.

After delayed transplantation, MSC induced a decreased expression of genes related to external stimulus and vitamin and nutrient levels response at both time post-grafting (Fig. 3A and B), and among them Alpl and Fabp4 were the most down-regulated genes (Fig. 7C). For OEC delayed transplantation, besides genes related to tissue repair, the genes involved in the response to external stimulus were potentiated at the early time after treatment (Fig. 3A), being Irf7 the most relevant gene in this functional group (Fig. 7D).

## Discussion

Several studies have shown that transplantation of MSC and of OEC after SCI exert beneficial effects on functional recovery, tissue protection, axonal regeneration and remyelination. However, the mechanisms and changes triggered by the grafted cells on the spinal cord are still poorly understood. The results of the present study contribute to the knowledge of the role played by MSC and OEC transplants in the injured spinal cord, and provide important information regarding the genes and pathways modified by these cells when grafted into the injured spinal cord.

### Cell Therapy and Tissue Healing

Endogenous repair or wound healing processes are activated quickly, synchronically and sequentially after tissue damage in order to avoid the expansion of the injury and to provide a suitable environment for tissue regeneration [43], [44]. The repair dynamic events include: blood clotting and fibrin formation, recruitment of inflammatory cells, angiogenesis, ECM synthesis and collagen deposition, and cell proliferation [43], [44]. All these events have to be precisely regulated, otherwise they can induce undesirable effects in the damaged tissue, such as cyst formation, tissue fibrosis and prevention of regenerative mechanisms [44]. Chronification of the wound healing process occurs after injuries to the CNS, and thus, therapies aimed at inducing the remission of these events are expected to induce neuroprotection and a better milieu for axonal regeneration. The results showed herein suggest that both MSC and OEC can promote different aspects of tissue repair mechanisms, which may explain, in part, the beneficial effects of their grafting after injury.

Our microarray data showed that acute transplantation of both cell types induced early gene expression related to regulation of cell adhesion, hemopoiesis, response to hypoxia, organ development and ECM organization. These changes demonstrate shared mechanisms of MSC and OEC transplants, probably by increasing some aspects of the wound response. However, other genes associated to ECM organization, blood vessel development, cell adhesion and response to wounding and tissue development were up-regulated only by acute MSC transplant at early times, indicative that the changes in these processes were more important after MSC than OEC acute injection. On the other hand, when MSC and OEC were transplanted in subacute SCI, most of the effects induced by both cell grafts were related to the suppression of biological events, especially, mechanisms related to tissue repair. Nevertheless, the enriched pathways shared by both cells were limited since those related to tissue repair were decreased specifically by MSC or OEC treatment. Thus, a reduction of organ development, response to nutrient levels, blood vessel development and ECM organization events were observed during the early time by MSC, whereas blood vessel development and ECM organization were suppressed only by OEC at later time. Therefore, MSC or OEC transplantation may induce changes in mechanisms of tissue repair by both common and specific events and in a complex temporal pattern. Moreover, these changes seem to be more important after MSC transplant, probably because their role during tissue repair [45], [46], [47].

#### ECM remodeling after cell transplantation

As previously mentioned, cell therapy induced changes in genes related to ECM organization, which is necessary for effective tissue repair [43], [44]. Both acute MSC and OEC grafts enhanced the expression of genes related to ECM organization and collagen synthesis, in particular those related with collagen I deposition, such as the genes coding for collagen type I (Col1a1 and Col1a2), and collagen type VIII (Col8a1), Plod2 (procollagen-lysine, 2-oxoglutarate 5-dioxygenase 2), a lysyl hydroxylase enzyme that influences the stability of intermolecular collagen cross-links [48], and the matrix metalloproteases Mmp13, Mmp12, Mmp8 and Mmp9. Fibril collagen deposition, specially of collagen type I and III, is essential during the early phase of tissue repair and wound healing [43], forming the base of the new ECM that replaces the fibrin clot and allows the regeneration of the tissue. After SCI, collagen I increases and is distributed around blood vessels forming a scaffold for angiogenesis [49]. The beneficial effects of both MSC [15] and OEC [33] have been related with the formation of new blood vessels. In fact, the results of the microarray showed an enrichment of blood vessel development events after acute MSC transplantation. Furthermore, collagen type VIII is related with vascular smooth muscle cells, promoting their migration and growth [50]. In addition, Plod2 is involved in the collagen fibril deposition [48] and its expression is increased in response to hypoxia [51]. Matrix metalloproteases also play an important role during tissue repair participating in matrix degradation to allow debriding, ECM restyling, and cell migration. Mmp13, also known as collagenase-3, is involved in angiogenesis during tissue repair [52] and in the vascularization of tumors [53]. Moreover, it was recently demonstrated that Mmp13 is essential for granulated tissue formation in wound healing [54]. In the CNS, Mmp13 expression is induced in astrocytes by hypoxia signaling and increases the permeability of the blood-brain-barrier [55]. Thus, Mmp13 and other matrix metalloproteases play an important role in vascular physiology regulation [56]. Other important protein in the ECM remodeling that we found up-regulated after acute transplants is Postn (periostin). Periostin is an ECM protein that interacts with other matrix components, such as collagen I [57]. Their interaction allows the activation of LOX (lysyl oxidase) [57], an enzyme responsible for cross-linking other proteins with collagen. It is important to note that the overexpression of most of the above mentioned genes was higher after MSC than OEC transplant, probably indicating stronger effects of the MSC on ECM remodeling.

Regarding delayed transplants, the genes related to collagen type I and Plod2 were not changed, whereas the expression of Col8a1 and Lox were reduced by both cell treatments, although with temporal differences. Genes coding for matrix metalloproteases, such as Mmp12, Mmp8 and Mmp9, were reduced by MSC grafting and increased by OEC grafting, reflecting different regulatory mechanisms of the ECM between MSC and OEC. All together, the regulation of ECM remodeling seems to be achieved by mechanisms shared by both MSC and OEC during the acute phase after SCI, while delayed injection of the cells induces different genetic changes. Furthermore, most of the ECM related genes modulated by the cells are involved in vascular physiology and angiogenesis suggesting a role of transplanted cells in revascularization of the damaged tissue.

#### MSC and tissue repair

Our data suggest a regulatory role for MSC when transplanted into the injured spinal cord, boosting the restorative mechanisms at early phases following SCI but inducing resolution at later time points and accelerating the homeostasis of the injured tissue. The potentiation of repair processes could be the most probable mechanism underlying the beneficial effects of the MSC transplantation after SCI regarding tissue sparing [12], [15], [29], [52], neoangiogenesis [15] and inflammatory modulation [27], [28]. *In vivo*, MSC enhance the regenerative potential of different tissues as a result of paracrine mechanisms that become activated when the cells are exposed to an injury environment [45], [46], [47], [58], [59]. During the inflammation phase of wound healing, proinflammatory mediators can activate regulatory functions in MSC, leading to secretion of inflammatory mediators [60] and anti-inflammatory cytokines [61]. As a result, the activity of MSC in the injured tissues potentiates wound healing and tissue regeneration [62], [63]. During the proliferation phase, paracrine factors secreted by the MSC, including bFGF, VEGF, HGF, IL10 and MMP-9 [64], [65], stimulate survival and proliferation of resident cells [66], [67], angiogenesis [68], [69] and vascular stability [70]. Moreover, it has been proposed that perivascular cells named pericytes are indeed MSC that readily infiltrate tissues after blood vessel breakdown, acting as a cellular sensor of damage and secreting mediators for tissue repair [45]. Pericytes have been found to play a crucial role in repair processes after SCI [71]. This can explain, in part, the potential benefits of the therapeutic transplantation of MSC [58], [72], [73].

#### OEC and tissue repair

As discussed above, the acute presence of OEC in the injured spinal cord induced the expression of genes related to tissue repair, whereas a reduction of some of these pathways was observed after delayed transplantation. Of interest, most of the early changes observed after acute OEC injection were related to genes associated with inflammatory and immune response, particularly Cxcl9 and Nos2 (inducible Nitric oxide synthase 2 or iNos). Nos2 expression after SCI is closely related to macrophage/microglia activation, but it is also up-regulated in neurons, astrocytes and oligodendrocytes [74]. These results may be related to previous data from our laboratory describing earlier and stronger recruitment of microglia/macrophages by an acute OEC graft in the injured spinal cord of rats [33]. In addition, administration of Nos2 inhibitors reduced tissue sparing, functional outcomes and angiogenic activity of OEC transplantation after SCI [75], suggesting a beneficial role of the Nos2 overexpression induced by OEC. In agreement with our results, gene expression analysis has shown that OEC express a large range of genes involved in wound healing, ECM remodeling, cell adhesion and angiogenesis [32]. Despite the marked early changes in expression profile induced by acute OEC transplant following SCI, few changes were observed at 7 days following the acute transplantation. This could be due to rejection of the transplanted cells in the spinal cord, as well as to poor integration and migration but strong boundering [76], [77] of the OEC in the injured spinal cord. Delayed transplantation of OEC after SCI induced a few changes in the gene expression profile. Among the modulated mechanisms, we found reduced expression of tissue repair associated genes, suggesting a modulatory capability of OEC when transplanted in a subacute SCI.

The results showed herein suggest that the early presence of MSC or OEC in the injured spinal cord may potentiate or accelerate some of the tissue repair mechanisms. This could enable a faster homeostasis recovery, enhancing ECM remodeling, limiting expansion of the lesion and creating a favorable environment for tissue regeneration, while at delayed times the transplanted cells seem to reduce the tissue repair response to inhibit chronification of the wound healing process.

### Grafted Cell Rejection

One of the most remarkable results observed after acute or delayed cell transplantation was the up-regulation of genes involved in immune response and specifically the response against foreign organisms. Indeed, the gene expression profile was enriched in the cluster of processes such as immune and defense response, leukocyte activation, chemotaxis and migration, leukocyte mediated immunity, antigen processing and presentation, and adaptive immune response. In the list of genes associated with these processes we found an overexpression of the rat major histocompatibility complex (MHC) class I, such as RT1-N1, RT1-N3 and RT1-EC2, and class II, such as RT1-Da and RT1-Ba (Fig. S2) [78], [79]. The expression of these genes may indicate the presence of antigen presenting cells (APC), presenting MHC class I and class II antigens. The presence of MHC class I antigens stimulates the immature CD8+ lymphocytes to become mature cytotoxic T lymphocytes [80], [81], [82] that induce apoptosis in the grafts. In our gene array we found an over expression of CD8 indicating the presence of mature or immature cytotoxic T lymphocytes in the injured spinal cord tissue after transplantation. In addition, the MHC class II antigen presented by APC interacts with CD4+ immature lymphocytes inducing their maturation to mature T-helper CD4+ lymphocytes [82]. We also found an up-regulation of CD74, involved in the formation and transport to membrane of MHC class II, CD4 and some chemokines that act as chemoattractants to T-cells, such as Cxcl9 and Cxcl10 (Fig. S2) [83], [84], [85], and B-cells, such as Cxcl13 [84], [85], suggesting recruitment of CD4+ T-cells and B-cells. Collectively, these results may indicate a host rejection response against the grafted cells initiated at least at day 7 post-transplantation of MSC and OEC. Although this event was found after both MSC and OEC transplants, the earlier increase of CD8 and CD4 induced by acute OEC than MSC injection and the higher overexpression of MHC I and II after delayed MSC than OEC injection suggest a differential rejection response.

Some published data point to limited survival of grafted cells after spinal cord contusion. Reaction against engrafted MSC was observed in the intact spinal cord [86], [87], with an important recruitment of macrophages, leading to a marked reduction in the number of cells between 2 and 4 weeks after their injection in comparison to a syngeneic transplant [86]. Furthermore, the administration of cyclosporine A, an immunosuppressant drug, prolongs the survival of the grafted cells [86], [87], confirming the immune rejection induced in the spinal cord. After SCI, limited survival was also found of xenogeneic human MSC [12] and allogeneic MSC grafted in contused rats [27], [88] and of allografts in dogs [89]. In the same way, OEC transplanted after contusion injury in rats also had reduced survival [24]. Survival of the grafted cells largely influences the treatment success, and thus, the combination of cell grafts with immunosuppressant agents is probably needed to optimize the efficacy of cell therapies.

### Conclusion

Our data provide an overview of the mechanisms modulated by MSC and OEC when transplanted after SCI. The changes observed in gene expression profile suggest that transplantation of both, MSC and OEC, accelerates and/or enhances tissue repair events during the early stages of the injury, while it tends to resolve these processes at later time points. However, the activation of the adaptive immune response in the grafted spinal cord after transplantation is indicative of cell rejection. Further investigations are needed to better understand the neuroprotective actions of MSC and OEC grafts, as well as to avoid cell rejection to optimize cell therapies for clinical application in SCI.

## Supporting Information

Figure S1 **Microarray data validation.** (A) *In silico* comparison of our gene array results showed a high concordance of up-regulated genes and down-regulated genes with results previously published after the same type of injury in rats (GSE464) and in mice (GSE5296), but not with another type of SCI in rats (GSE22161). (B) The validation of some target genes by RT-PCR indicated good concordance between expression changes obtained by microarray and by RT-PCR (Pearson correlation, r = 0.87, p<0.0001). In this comparison a few discrepancies were observed but only in target genes that did not reach the cut-off of significant changes (inside the square in B).
(TIF)

Figure S2 **Rejection response related genes.** Graphical representation of RT1-N1, RT1-N3, RT1-Ba and Cxcl10 genes. Each graph shows the fold changes of the corresponding gene after acute (2 and 7 days post injury) and delayed (9 and 14 days post injury) cell transplantation in comparison to vehicle value. Data are represented as mean ± SEM. $ p<0.05 OEC vs. vehicle, & p<0.05 MSC vs. vehicle.
(TIF)

Table S1 **Primers used for RT-PCR and microarray validation.** RT-PCR was performed with primers of genes with expression changed at least in one experimental condition of the study.
(DOC)

Table S2 **Functional annotation cluster: MSC 0.2 UP.**
(DOC)

Table S3 **Functional annotation cluster: OEC 0.2 UP.**
(DOC)

Table S4 **Functional annotation cluster: MSC and OEC 0.2 UP.**
(DOC)

Table S5 **Functional annotation cluster: MSC 0.2 Down.**
(DOC)

Table S6 **Functional annotation cluster: MSC 0.7 UP.**
(DOC)

Table S7 **Functional annotation cluster: OEC 0.7 UP.**
(DOC)

Table S8 **Functional annotation cluster: MSC and OEC 0.7 UP.**
(DOC)

Table S9 **Functional annotation cluster: MSC 0.7 DOWN.**
(DOC)

Table S10 **Functional annotation cluster: MSC and OEC 0.7 DOWN.**
(DOC)

Table S11 **Functional annotation cluster: MSC 7.2 UP.**
(DOC)

Table S12 **Functional annotation cluster: OEC 7.2 UP.**
(DOC)

Table S13 **Functional annotation cluster: MSC 7.2 DOWN.**
(DOC)

Table S14 **Functional annotation cluster: MSC and OEC 7.2 DOWN.**
(DOC)

Table S15 **Functional annotation cluster: MSC 7.7 UP.**
(DOC)

Table S16 **Functional annotation cluster: OEC 7.7 UP.**
(DOC)

Table S17 **Functional annotation cluster: MSC and OEC 7.7 UP.**
(DOC)

Table S18 **Functional annotation cluster: MSC 7.7 DOWN.**
(DOC)

Table S19 **Functional annotation cluster: OEC 7.7 DOWN.**
(DOC)

Table S20 **Functional annotation cluster: MSC and OEC 7.7 DOWN.**
(DOC)
