# Supplementary figures and images for: Gene Expression Changes in the Injured Spinal Cord Following Transplantation of Mesenchymal Stem Cells or Olfactory Ensheathing Cells
Source: PLoS One. 2013 Oct 11;8(10):e76141. doi: 10.1371/journal.pone.0076141 (PMC3795752; doi:10.1371/journal.pone.0076141)

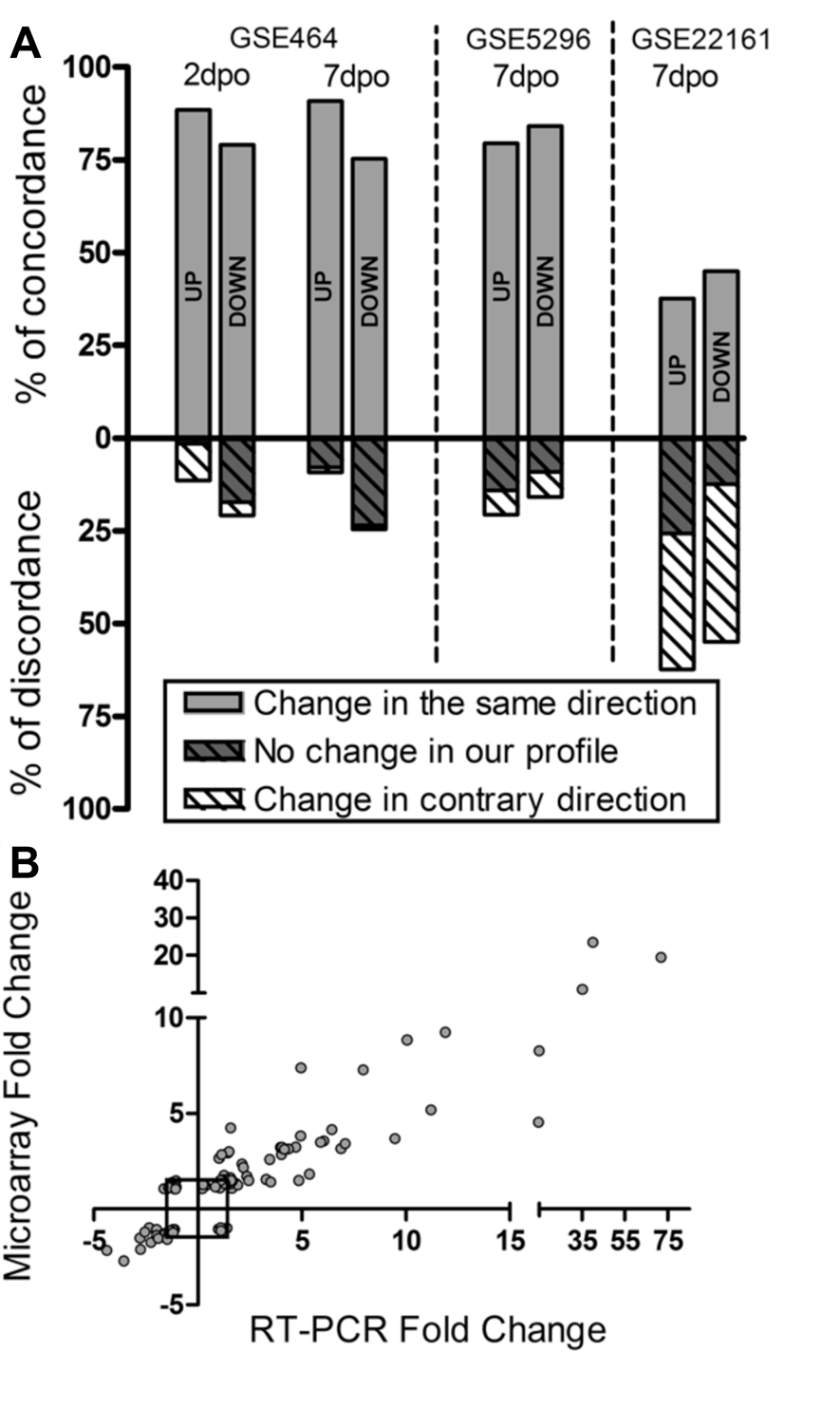

Supplement: Figure S1 — Microarray data validation. (A) In silico comparison of our gene array results showed a high concordance of up-regulated genes and down-regulated genes with results previously published after the same type of injury in rats (GSE464) and in mice (GSE5296), but not with another type of SCI in rats (GSE22161). (B) The validation of some target genes by RT-PCR indicated good concordance between expression changes obtained by microarray and by RT-PCR (Pearson correlation, r = 0.87, p<0.0001). In this comparison a few discrepancies were observed but only in target genes that did not reach the cut-off of significant changes (inside the square in B). (TIF) [file pone.0076141.s001.tif]

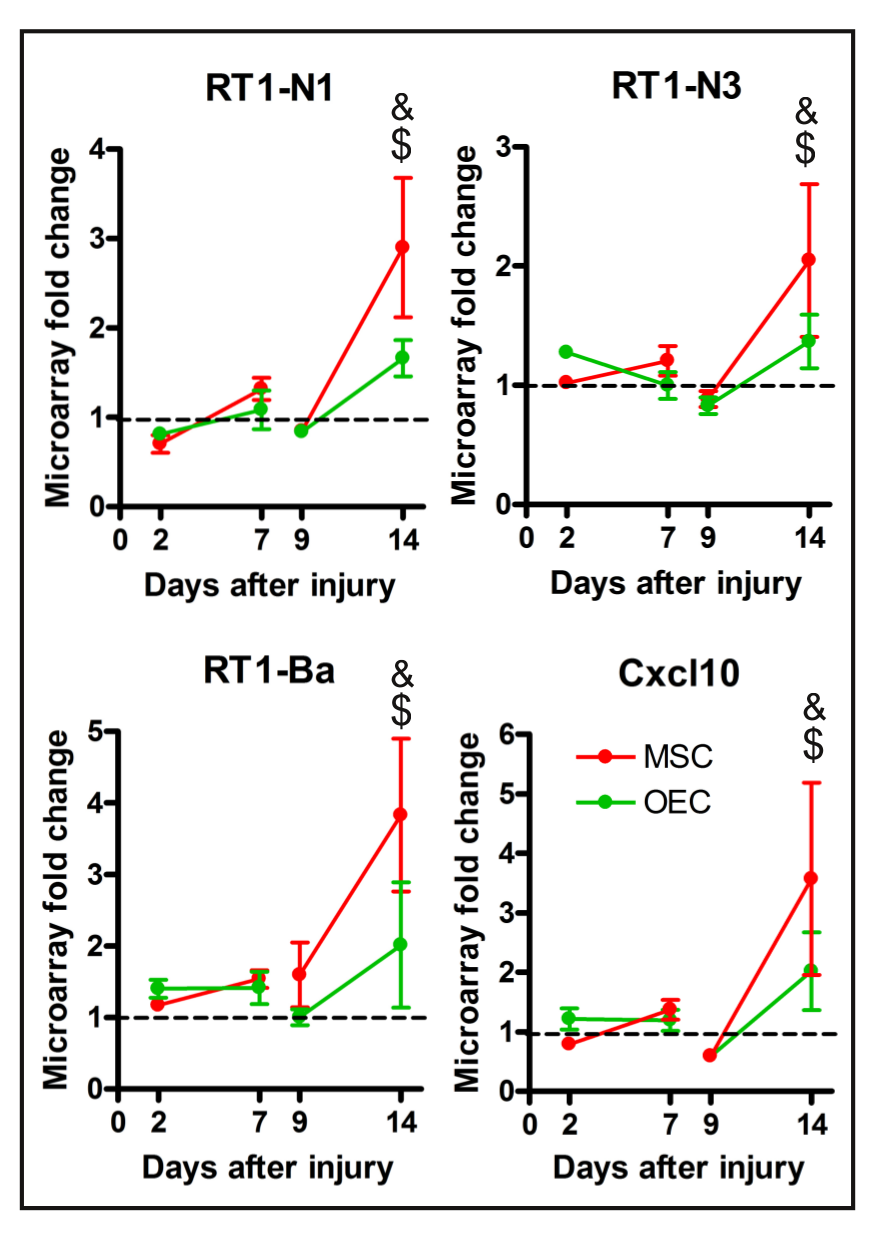

Supplement: Figure S2 — Rejection response related genes. Graphical representation of RT1-N1, RT1-N3, RT1-Ba and Cxcl10 genes. Each graph shows the fold changes of the corresponding gene after acute (2 and 7 days post injury) and delayed (9 and 14 days post injury) cell transplantation in comparison to vehicle value. Data are represented as mean ± SEM. $ p<0.05 OEC vs. vehicle, & p<0.05 MSC vs. vehicle. (TIF) [file pone.0076141.s002.tif]
