# Supplementary material for: Gene Expression Changes in the Injured Spinal Cord Following Transplantation of Mesenchymal Stem Cells or Olfactory Ensheathing Cells
Source: PLoS One. 2013 Oct 11;8(10):e76141. doi: 10.1371/journal.pone.0076141 (PMC3795752; doi:10.1371/journal.pone.0076141)
Supplement: Table S1 — Primers used for RT-PCR and microarray validation. RT-PCR was performed with primers of genes with expression changed at least in one experimental condition of the study. (DOC) [file pone.0076141.s003.doc]

| **Table S1. Primers used for RT-PCR and microarray validation. RT-PCR was performed with primers of genes with expression changed at least in one experimental condition of the study** | |
| --- | --- |
| **Mmp13** | Forward: ...GAAGATGTCAGGCATAAAGG... (21bp) |
|  | Reverse: ...TTCTCCATCTCTGTGTCCTC... (20bp) |
| **Hgf** | Forward: ...GGCATTCCAACACAAACAAC... (20bp) |
|  | Reverse: ...ATCTGTTTGCGTTTCTCCTC... (20bp) |
| **Lcn2** | Forward: ...GCGAATGCGGTCCAGAAAG... (19bp) |
|  | Reverse: ...CCTGACGAGGATGGAAGTG... (19bp) |
| **Plod2** | Forward: ...TGAGTGGCTCTTTGAGATGG... (20bp) |
|  | Reverse: ...TCCTCTCTCTTCTTCTTCAACC... (22bp) |
| **Bcl2** | Forward: ...GCCTTCTTTGAGTTCGGTG... (19bp) |
|  | Reverse: ...GCCAGGAGAAATCAAACAGAG... (21bp) |
| **Itgax** | Forward: ...GACGGAAGATACCCAGCAG... (19bp) |
|  | Reverse: ...AGTCATCTGTGAGCCTCC... (18bp) |
| **Scarb1** | Forward: ...CCCAGATGTCACACTGTCC... (19bp) |
|  | Reverse: ...GACGGAGAAAGTCAGGAGC... (19bp) |
| **Hif1a** | Forward: ...AGTGAACAGGATGGAATGGAG... (21bp) |
|  | Reverse: ...GGTTTCTGCTGCCTTGTATG... (20bp) |
| **Lpl** | Forward: ...GCTGGTGGGAAATGATGTG... (19bp) |
|  | Reverse: ...TCATCAGGAGAAAGGCGAC... (19bp) |

Code of the primers used in the RT-PCR experiments. For each selected gene the name of the gene, the forward and the reverse sequence and the length of the primer (pair of bases) are indicated.
