# Supplementary material for: Gene Expression Changes in the Injured Spinal Cord Following Transplantation of Mesenchymal Stem Cells or Olfactory Ensheathing Cells
Source: PLoS One. 2013 Oct 11;8(10):e76141. doi: 10.1371/journal.pone.0076141 (PMC3795752; doi:10.1371/journal.pone.0076141)
Supplement: Table S2 — Functional annotation cluster: MSC 0.2 UP. (DOC) [file pone.0076141.s004.doc]

| **Table S2. Functional annotation cluster: MSC 0.2 UP** | | | | | |
| --- | --- | --- | --- | --- | --- |
| **Functional annotation cluster (enriched score)** | **G** | **P Value** | **Functional annotation cluster (enriched score)** | **G** | **P Value** |
| **1. Extracellular matrix organization (3.17)** |  |  | GO:0010033~response to organic substance | 11 | 0.0052 |
| GO:0043062~extracellular structure organization | 6 | 2.58E-4 | GO:0046660~female sex differentiation | 4 | 0.0053 |
| GO:0030198~extracellular matrix organization | 5 | 4.43E-4 | GO:0007584~response to nutrient | 5 | 0.0112 |
| GO:0030199~collagen fibril organization | 3 | 0.0033 | GO:0008406~gonad development | 4 | 0.0132 |
| **2. Blood vessel development (2.07)** |  |  | GO:0045137~development of primary sexual characteristics | 4 | 0.0184 |
| GO:0048771~tissue remodeling | 4 | 0.0024 | GO:0007548~sex differentiation | 4 | 0.0273 |
| GO:0001568~blood vessel development | 6 | 0.0027 | GO:0048608~reproductive structure development | 4 | 0.0288 |
| GO:0001944~vasculature development | 6 | 0.0031 | GO:0031667~response to nutrient levels | 5 | 0.0312 |
| GO:0001974~blood vessel remodeling | 3 | 0.0037 | GO:0003006~reproductive developmental process | 5 | 0.0334 |
| GO:0060688~regulation of morphogenesis of a branching structure | 3 | 0.0048 | GO:0009991~response to extracellular stimulus | 5 | 0.0389 |
| GO:0048514~blood vessel morphogenesis | 5 | 0.0076 | GO:0046545~development of primary female sexual characteristics | 3 | 0.0426 |
| GO:0051239~regulation of multicellular organismal process | 11 | 0.0083 | **5. Response to wounding and tissue development (1.88)** |  |  |
| GO:0009653~anatomical structure morphogenesis | 11 | 0.015 | GO:0009611~response to wounding | 7 | 0.0089 |
| GO:0048513~organ development | 14 | 0.0163 | GO:0008544~epidermis development | 4 | 0.0092 |
| GO:0048731~system development | 16 | 0.0224 | GO:0007398~ectoderm development | 4 | 0.0117 |
| GO:0050793~regulation of developmental process | 8 | 0.0269 | GO:0009888~tissue development | 8 | 0.0155 |
| GO:0048856~anatomical structure development | 16 | 0.0357 | GO:0001501~skeletal system development | 5 | 0.0242 |
| **3. Cell adhesion (2.03)** |  |  | **6. Negative regulation of response to stimulus (1.71)** |  |  |
| GO:0022610~biological adhesion | 7 | 0.0091 | GO:0048585~negative regulation of response to stimulus | 4 | 0.0095 |
| GO:0007155~cell adhesion | 7 | 0.0091 | GO:0048583~regulation of response to stimulus | 6 | 0.0385 |
| **4. Response to nutrient (1.89)** |  |  | **7. Response to monosaccharide stimulus (1.37)** |  |  |
| GO:0033273~response to vitamin | 5 | 0.0012 | GO:0034284~response to monosaccharide stimulus | 3 | 0.0425 |
| GO:0009605~response to external stimulus | 11 | 0.0019 | GO:0009746~response to hexose stimulus | 3 | 0.0425 |
| Continue in the next column |  |  |  |  |  |

Results of the functional annotation clustering performed using the DAVID's platform. Below each functional cluster (gray boxes) the GO clustered term (left columns), the number of differentially expressed genes that were present in each GO term (G, middle columns) and the statistical p value of GO term enrichment are indicated.
