# Supplementary material for: Gene Expression Changes in the Injured Spinal Cord Following Transplantation of Mesenchymal Stem Cells or Olfactory Ensheathing Cells
Source: PLoS One. 2013 Oct 11;8(10):e76141. doi: 10.1371/journal.pone.0076141 (PMC3795752; doi:10.1371/journal.pone.0076141)
Supplement: Table S3 — Functional annotation cluster: OEC 0.2 UP. (DOC) [file pone.0076141.s005.doc]

| **Table S3. Functional annotation cluster: OEC 0.2 UP** | | | | | |
| --- | --- | --- | --- | --- | --- |
| **Functional annotation cluster (enriched score)** | **G** | **P Value** | **Functional annotation cluster (enriched score)** | **G** | **P Value** |
| **1. Immune response (2.23)** | 8 | 0.0002 | GO:0031399~regulation of protein modification process | 5 | 0.0112 |
| GO:0006955~immune response | 7 | 0.0017 | GO:0009057~macromolecule catabolic process | 6 | 0.0123 |
| GO:0009611~response to wounding | 12 | 0.0019 | GO:0051436~negative regulation of ubiquitin-protein ligase activity during mitotic cell cycle | 3 | 0.0127 |
| GO:0006950~response to stress | 8 | 0.0052 | GO:0031145~anaphase-promoting complex-dependent proteasomal ubiquitin-dependent protein catabolic process | 3 | 0.0127 |
| GO:0002376~immune system process | 6 | 0.0065 | GO:0051444~negative regulation of ubiquitin-protein ligase activity | 3 | 0.0131 |
| GO:0006952~defense response | 8 | 0.0109 | GO:0051352~negative regulation of ligase activity | 3 | 0.0131 |
| GO:0009605~response to external stimulus | 8 | 0.0232 | GO:0006511~ubiquitin-dependent protein catabolic process | 4 | 0.0136 |
| GO:0010646~regulation of cell communication | 4 | 0.0283 | GO:0051603~proteolysis involved in cellular protein catabolic process | 5 | 0.0141 |
| GO:0006954~inflammatory response | 18 | 0.0474 | GO:0044257~cellular protein catabolic process | 5 | 0.0146 |
| GO:0050896~response to stimulus | 8 | 0.0002 | GO:0051439~regulation of ubiquitin-protein ligase activity during mitotic cell cycle | 3 | 0.0150 |
| **2. Biological regulation (2.03)** |  |  | GO:0031397~negative regulation of protein ubiquitination | 3 | 0.0165 |
| GO:0065007~biological regulation | 26 | 0.0033 | GO:0030163~protein catabolic process | 5 | 0.0177 |
| GO:0050789~regulation of biological process | 24 | 0.0071 | GO:0051438~regulation of ubiquitin-protein ligase activity | 3 | 0.0180 |
| GO:0048518~positive regulation of biological process | 13 | 0.0086 | GO:0051340~regulation of ligase activity | 3 | 0.0191 |
| GO:0050794~regulation of cellular process | 21 | 0.0368 | GO:0010498~proteasomal protein catabolic process | 3 | 0.0272 |
| **3. Catabolic and protein metabolic process (1.81)** |  |  | GO:0043161~proteasomal ubiquitin-dependent protein catabolic process | 3 | 0.0272 |
| GO:0009056~catabolic process | 9 | 0.0044 | GO:0031396~regulation of protein ubiquitination | 3 | 0.0317 |
| GO:0044248~cellular catabolic process | 8 | 0.0046 | GO:0043086~negative regulation of catalytic activity | 4 | 0.0349 |
| GO:0006508~proteolysis | 8 | 0.0070 | GO:0032268~regulation of cellular protein metabolic process | 5 | 0.0394 |
| GO:0044265~cellular macromolecule catabolic process | 6 | 0.0072 | GO:0031400~negative regulation of protein modification process | 3 | 0.0437 |
| Continue in the next column |  |  |  |  |  |

Results of the functional annotation clustering performed using the DAVID's platform. Below each functional cluster (gray boxes) the GO clustered term (left columns), the number of differentially expressed genes that were present in each GO term (G, middle columns) and the statistical p value of GO term enrichment are indicated.
