# Supplementary material for: Gene Expression Changes in the Injured Spinal Cord Following Transplantation of Mesenchymal Stem Cells or Olfactory Ensheathing Cells
Source: PLoS One. 2013 Oct 11;8(10):e76141. doi: 10.1371/journal.pone.0076141 (PMC3795752; doi:10.1371/journal.pone.0076141)
Supplement: Table S4 — Functional annotation cluster: MSC and OEC 0.2 UP. (DOC) [file pone.0076141.s006.doc]

| **Table S4. Functional annotation cluster: MSC and OEC 0.2 UP** | | | | | |
| --- | --- | --- | --- | --- | --- |
| **Functional annotation cluster (enriched score)** | **G** | **P Value** | **Functional annotation cluster (enriched score)** | **G** | **P Value** |
| **1. Regulation of cell adhesion (2.62)** |  |  | GO:0006954~inflammatory response | 5 | 0.0061 |
| GO:0030155~regulation of cell adhesion | 5 | 0.0006 | GO:0033273~response to vitamin | 4 | 0.0064 |
| GO:0010810~regulation of cell-substrate adhesion | 3 | 0.0087 | GO:0043010~camera-type eye development | 4 | 0.0064 |
| **2. Hemopoiesis (2.57)** |  |  | GO:0060541~respiratory system development | 4 | 0.0069 |
| GO:0002376~immune system process | 10 | 0.0005 | GO:0048729~tissue morphogenesis | 5 | 0.0071 |
| GO:0048534~hemopoietic or lymphoid organ development | 6 | 0.0015 | GO:0001568~blood vessel development | 5 | 0.0074 |
| GO:0002520~immune system development | 6 | 0.0019 | GO:0001822~kidney development | 4 | 0.0078 |
| GO:0030097~hemopoiesis | 5 | 0.0073 | GO:0001944~vasculature development | 5 | 0.0082 |
| GO:0006955~immune response | 6 | 0.0118 | GO:0009653~anatomical structure morphogenesis | 10 | 0.0099 |
| **3. Response to hypoxia (2.36)** |  |  | GO:0021536~diencephalon development | 3 | 0.0100 |
| GO:0001666~response to hypoxia | 5 | 0.0039 | GO:0048593~camera-type eye morphogenesis | 3 | 0.0100 |
| GO:0070482~response to oxygen levels | 5 | 0.0048 | GO:0008284~positive regulation of cell proliferation | 6 | 0.0106 |
| **4. Organ development (2.27)** |  |  | GO:0001501~skeletal system development | 5 | 0.0116 |
| GO:0048513~organ development | 18 | 9.89E-06 | GO:0001654~eye development | 4 | 0.0122 |
| GO:0048731~system development | 18 | 0.0002 | GO:0043627~response to estrogen stimulus | 4 | 0.0132 |
| GO:0009605~response to external stimulus | 11 | 0.0003 | GO:0009611~response to wounding | 6 | 0.0142 |
| GO:0048856~anatomical structure development | 18 | 0.0005 | GO:0009790~embryonic development | 7 | 0.0157 |
| GO:0007584~response to nutrient | 6 | 0.0006 | GO:0048545~response to steroid hormone stimulus | 5 | 0.0167 |
| GO:0060324~face development | 3 | 0.0009 | GO:0042221~response to chemical stimulus | 16 | 0.0205 |
| GO:0009888~tissue development | 9 | 0.0010 | GO:0033189~response to vitamin A | 3 | 0.0212 |
| GO:0007275~multicellular organismal development | 18 | 0.0011 | GO:0048592~eye morphogenesis | 3 | 0.0238 |
| GO:0060322~head development | 3 | 0.0014 | GO:0042127~regulation of cell proliferation | 7 | 0.0256 |
| GO:0006950~response to stress | 13 | 0.0014 | GO:0009612~response to mechanical stimulus | 3 | 0.0271 |
| GO:0051216~cartilage development | 4 | 0.0016 | GO:0035270~endocrine system development | 3 | 0.0278 |
| GO:0001655~urogenital system development | 5 | 0.0020 | GO:0050794~regulation of cellular process | 23 | 0.0323 |
| GO:0031667~response to nutrient levels | 6 | 0.0026 | GO:0048732~gland development | 4 | 0.0326 |
| GO:0050896~response to stimulus | 23 | 0.0027 | GO:0010033~response to organic substance | 8 | 0.0366 |
| GO:0048518~positive regulation of biological process | 15 | 0.0028 | GO:0006952~defense response | 5 | 0.0430 |
| GO:0048522~positive regulation of cellular process | 14 | 0.0032 | GO:0009887~organ morphogenesis | 6 | 0.0437 |
| GO:0009991~response to extracellular stimulus | 6 | 0.0035 | **5. Extracellular matrix organization (2.24)** |  |  |
| GO:0032502~developmental process | 18 | 0.0035 | GO:0030198~extracellular matrix organization | 4 | 0.0030 |
| GO:0021983~pituitary gland development | 3 | 0.0060 | GO:0043062~extracellular structure organization | 4 | 0.0109 |
| Continue in the next column |  |  |  |  |  |

Results of the functional annotation clustering performed using the DAVID's platform. Below each functional cluster (gray boxes) the GO clustered term (left columns), the number of differentially expressed genes that were present in each GO term (G, middle columns) and the statistical p value of GO term enrichment are indicated.
