# Supplementary material for: Gene Expression Changes in the Injured Spinal Cord Following Transplantation of Mesenchymal Stem Cells or Olfactory Ensheathing Cells
Source: PLoS One. 2013 Oct 11;8(10):e76141. doi: 10.1371/journal.pone.0076141 (PMC3795752; doi:10.1371/journal.pone.0076141)
Supplement: Table S6 — Functional annotation cluster: MSC 0.7 UP. (DOC) [file pone.0076141.s008.doc]

| **Table S6. Functional annotation cluster: MSC 0.7 UP** | | | | | |
| --- | --- | --- | --- | --- | --- |
| **Functional annotation cluster (enriched score)** | **G** | **P Value** | **Functional annotation cluster (enriched score)** | **G** | **P Value** |
| **1. Immune response (3.02)** | 12 | 2.03E-07 | GO:0002694~regulation of leukocyte activation | 4 | 0.0233 |
| GO:0006955~immune response | 13 | 7.41E-06 | GO:0050865~regulation of cell activation | 4 | 0.0261 |
| GO:0002376~immune system process | 10 | 8.62E-06 | GO:0051094~positive regulation of developmental process | 5 | 0.0296 |
| GO:0002682~regulation of immune system process | 10 | 1.14E-05 | GO:0002253~activation of immune response | 3 | 0.0408 |
| GO:0006952~defense response | 8 | 1.38E-05 | GO:0002526~acute inflammatory response | 3 | 0.0417 |
| GO:0006954~inflammatory response | 10 | 2.27E-05 | **2. Leukocyte migration (2.21)** |  |  |
| GO:0009611~response to wounding | 13 | 3.04E-05 | GO:0007626~locomotory behavior | 6 | 0.0010 |
| GO:0009605~response to external stimulus | 16 | 0.0001 | GO:0042330~taxis | 4 | 0.0037 |
| GO:0006950~response to stress | 19 | 0.0001 | GO:0006935~chemotaxis | 4 | 0.0037 |
| GO:0048518~positive regulation of biological process | 7 | 0.0002 | GO:0007610~behavior | 7 | 0.0046 |
| GO:0048584~positive regulation of response to stimulus | 7 | 0.0002 | GO:0050900~leukocyte migration | 3 | 0.0197 |
| GO:0002684~positive regulation of immune system process | 16 | 0.0005 | GO:0040011~locomotion | 5 | 0.0427 |
| GO:0007165~signal transduction | 16 | 0.0009 | **3. Regulation of signal transduction (1.75)** |  |  |
| GO:0048522~positive regulation of cellular process | 30 | 0.0012 | GO:0009966~regulation of signal transduction | 9 | 0.0059 |
| GO:0050789~regulation of biological process | 8 | 0.0012 | GO:0010646~regulation of cell communication | 10 | 0.0074 |
| GO:0048583~regulation of response to stimulus | 6 | 0.0012 | GO:0010627~regulation of protein kinase cascade | 5 | 0.0133 |
| GO:0050776~regulation of immune response | 31 | 0.0019 | GO:0009967~positive regulation of signal transduction | 5 | 0.0222 |
| GO:0065007~biological regulation | 4 | 0.0020 | GO:0010740~positive regulation of protein kinase cascade | 4 | 0.0257 |
| GO:0002250~adaptive immune response | 4 | 0.0020 | GO:0010647~positive regulation of cell communication | 5 | 0.0321 |
| GO:0002460~adaptive immune response based on somatic recombination of immune receptors built from immunoglobulin superfamily domains | 28 | 0.0025 | GO:0043122~regulation of I-kappaB kinase/NF-kappaB cascade | 3 | 0.0453 |
| GO:0050794~regulation of cellular process | 4 | 0.0029 | **4. Negative regulation of apoptosis (1.62)** |  |  |
| GO:0002443~leukocyte mediated immunity | 4 | 0.0077 | GO:0042981~regulation of apoptosis | 8 | 0.0119 |
| GO:0002696~positive regulation of leukocyte activation | 4 | 0.0085 | GO:0043067~regulation of programmed cell death | 8 | 0.0128 |
| GO:0050867~positive regulation of cell activation | 4 | 0.0101 | GO:0010941~regulation of cell death | 8 | 0.0130 |
| GO:0002252~immune effector process | 23 | 0.0115 | GO:0048523~negative regulation of cellular process | 12 | 0.0165 |
| GO:0050896~response to stimulus | 3 | 0.0141 | GO:0051338~regulation of transferase activity | 5 | 0.0266 |
| GO:0016064~immunoglobulin mediated immune response | 4 | 0.0146 | GO:0048519~negative regulation of biological process | 12 | 0.0312 |
| GO:0050778~positive regulation of immune response | 3 | 0.0153 | GO:0043066~negative regulation of apoptosis | 5 | 0.0362 |
| GO:0019724~B cell mediated immunity | 3 | 0.0224 | GO:0043069~negative regulation of programmed cell death | 5 | 0.0379 |
| GO:0002449~lymphocyte mediated immunity | 4 | 0.0233 | GO:0060548~negative regulation of cell death | 5 | 0.0383 |
| Continue in the next column |  |  | GO:0050790~regulation of catalytic activity | 7 | 0.0424 |

Results of the functional annotation clustering performed using the DAVID's platform. Below each functional cluster (gray boxes) the GO clustered term (left columns), the number of differentially expressed genes that were present in each GO term (G, middle columns) and the statistical p value of GO term enrichment are indicated.
