# Supplementary material for: Gene Expression Changes in the Injured Spinal Cord Following Transplantation of Mesenchymal Stem Cells or Olfactory Ensheathing Cells
Source: PLoS One. 2013 Oct 11;8(10):e76141. doi: 10.1371/journal.pone.0076141 (PMC3795752; doi:10.1371/journal.pone.0076141)
Supplement: Table S8 — Functional annotation cluster: MSC and OEC 0.7 UP. (DOC) [file pone.0076141.s010.doc]

| **Table S8. Functional annotation cluster: MSC and OEC 0.7 UP** | | | | | |
| --- | --- | --- | --- | --- | --- |
| **Functional annotation cluster (enriched score)** | **G** | **P Value** | **Functional annotation cluster (enriched score)** | **G** | **P Value** |
| **1. Immune response (2.41)** |  |  | GO:0048583~regulation of response to stimulus | 5 | 0.0055 |
| GO:0006955~immune response | 7 | 3.68E-05 | GO:0032103~positive regulation of response to external stimulus | 3 | 0.0055 |
| GO:0002376~immune system process | 7 | 7.70E-04 | GO:0048584~positive regulation of response to stimulus | 4 | 0.0061 |
| GO:0032101~regulation of response to external stimulus | 4 | 0.0023 | GO:0050727~regulation of inflammatory response | 3 | 0.0076 |
| GO:0002682~regulation of immune system process | 5 | 0.0031 | GO:0050896~response to stimulus | 13 | 0.0081 |
| GO:0006950~response to stress | 8 | 0.0047 | GO:0031347~regulation of defense response | 3 | 0.0209 |
| Continue in the next column |  |  | GO:0050776~regulation of immune response | 3 | 0.0497 |

Results of the functional annotation clustering performed using the DAVID's platform. Below each functional cluster (gray boxes) the GO clustered term (left columns), the number of differentially expressed genes that were present in each GO term (G, middle columns) and the statistical p value of GO term enrichment are indicated.
