# Supplementary material for: Gene Expression Changes in the Injured Spinal Cord Following Transplantation of Mesenchymal Stem Cells or Olfactory Ensheathing Cells
Source: PLoS One. 2013 Oct 11;8(10):e76141. doi: 10.1371/journal.pone.0076141 (PMC3795752; doi:10.1371/journal.pone.0076141)
Supplement: Table S11 — Functional annotation cluster: MSC 7.2 UP. (DOC) [file pone.0076141.s013.doc]

| **Table S11. Functional annotation cluster: MSC 7.2 UP** | | | | | |
| --- | --- | --- | --- | --- | --- |
| **Functional annotation cluster (enriched score)** | **G** | **P Value** | **Functional annotation cluster (enriched score)** | **G** | **P Value** |
| **1. Immune response (3.2)** |  |  | GO:0002250~adaptive immune response | 3 | 0.0035 |
| GO:0006955~immune response | 10 | 4.80E-10 | GO:0002460~adaptive immune response based on somatic recombination of immune receptors built from immunoglobulin superfamily domains | 3 | 0.0035 |
| GO:0002376~immune system process | 10 | 6.29E-08 | GO:0002443~leukocyte mediated immunity | 3 | 0.0045 |
| GO:0050896~response to stimulus | 15 | 5.85E-06 | GO:0006935~chemotaxis | 3 | 0.0054 |
| GO:0006952~defense response | 7 | 6.82E-06 | GO:0042330~taxis | 3 | 0.0054 |
| GO:0006950~response to stress | 9 | 0.0001 | GO:0002682~regulation of immune system process | 4 | 0.0126 |
| GO:0002252~immune effector process | 4 | 0.0005 | GO:0050778~positive regulation of immune response | 3 | 0.0140 |
| GO:0009611~response to wounding | 5 | 0.0020 | GO:0007626~locomotory behavior | 3 | 0.0301 |
| GO:0048583~regulation of response to stimulus | 5 | 0.0023 | GO:0002684~positive regulation of immune system process | 3 | 0.0367 |
| GO:0006954~inflammatory response | 4 | 0.0027 | **2. Response to other organism (2.17)** |  |  |
| GO:0050776~regulation of immune response | 4 | 0.0027 | GO:0051704~multi-organism process | 5 | 0.0018 |
| GO:0002449~lymphocyte mediated immunity | 3 | 0.0030 | GO:0051707~response to other organism | 4 | 0.0045 |
| GO:0048584~positive regulation of response to stimulus | 4 | 0.0031 | GO:0009607~response to biotic stimulus | 4 | 0.0078 |
| GO:0009605~response to external stimulus | 6 | 0.0033 | GO:0009617~response to bacterium | 3 | 0.0320 |
| Continue in the next column |  |  |  | 5 | 0.0018 |

Results of the functional annotation clustering performed using the DAVID's platform. Below each functional cluster (gray boxes) the GO clustered term (left columns), the number of differentially expressed genes that were present in each GO term (G, middle columns) and the statistical p value of GO term enrichment are indicated.
