# Supplementary material for: Gene Expression Changes in the Injured Spinal Cord Following Transplantation of Mesenchymal Stem Cells or Olfactory Ensheathing Cells
Source: PLoS One. 2013 Oct 11;8(10):e76141. doi: 10.1371/journal.pone.0076141 (PMC3795752; doi:10.1371/journal.pone.0076141)
Supplement: Table S12 — Functional annotation cluster: OEC 7.2 UP. (DOC) [file pone.0076141.s014.doc]

| **Table S12. Functional annotation cluster: OEC 7.2 UP** | | |
| --- | --- | --- |
| **Functional annotation cluster (enriched score)** | **G** | **P Value** |
| 1. Response to external stimulus (2.32) |  |  |
| GO:0009605~response to external stimulus | 7 | 6.09E-4 |
| GO:0040007~growth | 3 | 0.0371 |

Results of the functional annotation clustering performed using the DAVID's platform. Below each functional cluster (gray boxes) the GO clustered term (left columns), the number of differentially expressed genes that were present in each GO term (G, middle columns) and the statistical p value of GO term enrichment are indicated
