# Supplementary material for: Gene Expression Changes in the Injured Spinal Cord Following Transplantation of Mesenchymal Stem Cells or Olfactory Ensheathing Cells
Source: PLoS One. 2013 Oct 11;8(10):e76141. doi: 10.1371/journal.pone.0076141 (PMC3795752; doi:10.1371/journal.pone.0076141)
Supplement: Table S13 — Functional annotation cluster: MSC 7.2 DOWN. (DOC) [file pone.0076141.s015.doc]

| **Table S13. Functional annotation cluster: MSC 7.2 DOWN** | | | | | |
| --- | --- | --- | --- | --- | --- |
| **Functional annotation cluster (enriched score)** | **G** | **P Value** | **Functional annotation cluster (enriched score)** | **G** | **P Value** |
| **1. Immune response (3.23)** |  |  | GO:0009719~response to endogenous stimulus | 8 | 0.0409 |
| GO:0006955~immune response | 11 | 0.0001 | GO:0050896~response to stimulus | 30 | 0.0413 |
| GO:0002376~immune system process | 12 | 0.0020 | **5. Negative regulation of cell differentiation (1.71)** |  |  |
| **2. Organ development (3.18)** |  |  | GO:0045596~negative regulation of cell differentiation | 6 | 0.0067 |
| GO:0032502~developmental process | 33 | 2.80E-06 | GO:0045778~positive regulation of ossification | 3 | 0.0092 |
| GO:0007275~multicellular organismal development | 31 | 3.24E-06 | GO:0051093~negative regulation of developmental process | 6 | 0.0141 |
| GO:0048731~system development | 26 | 0.0001 | GO:0051147~regulation of muscle cell differentiation | 3 | 0.0146 |
| GO:0048856~anatomical structure development | 26 | 0.0002 | GO:0045595~regulation of cell differentiation | 8 | 0.0231 |
| GO:0032501~multicellular organismal process | 39 | 0.0007 | GO:0050768~negative regulation of neurogenesis | 3 | 0.0380 |
| GO:0048513~organ development | 20 | 0.0012 | GO:0010721~negative regulation of cell development | 3 | 0.0406 |
| GO:0048646~anatomical structure formation involved in morphogenesis | 7 | 0.0101 | GO:0045786~negative regulation of cell cycle | 3 | 0.0458 |
| GO:0001944~vasculature development | 6 | 0.0105 | **6. Extracellular matrix organization (1.62)** |  |  |
| GO:0035295~tube development | 6 | 0.0126 | GO:0030198~extracellular matrix organization | 4 | 0.0132 |
| GO:0030154~cell differentiation | 16 | 0.0152 | GO:0043062~extracellular structure organization | 4 | 0.0423 |
| GO:0009790~embryonic development | 9 | 0.0193 | **7. Response to other organism (1.61)** |  |  |
| GO:0048869~cellular developmental process | 16 | 0.0209 | GO:0051707~response to other organism | 6 | 0.0147 |
| GO:0001525~angiogenesis | 4 | 0.0317 | GO:0009615~response to virus | 3 | 0.0274 |
| GO:0009653~anatomical structure morphogenesis | 12 | 0.0395 | GO:0051704~multi-organism process | 7 | 0.0274 |
| GO:0001568~blood vessel development | 5 | 0.0400 | GO:0009607~response to biotic stimulus | 6 | 0.0320 |
| **3. Negative regulation of cell communication (2.15)** |  |  | **8. Regulation of inlfammatory response (1.54)** |  |  |
| GO:0009968~negative regulation of signal transduction | 6 | 0.0052 | GO:0050727~regulation of inflammatory response | 4 | 0.0102 |
| GO:0010648~negative regulation of cell communication | 6 | 0.0093 | GO:0048583~regulation of response to stimulus | 7 | 0.0375 |
| **4. Response to external stimulus (1.85)** |  |  | GO:0031347~regulation of defense response | 4 | 0.0408 |
| GO:0009605~response to external stimulus | 13 | 0.0018 | GO:0048584~positive regulation of response to stimulus | 5 | 0.0411 |
| GO:0051240~positive regulation of multicellular organismal process | 7 | 0.0027 | **9. Bone development (1.54)** |  |  |
| GO:0045995~regulation of embryonic development | 3 | 0.0035 | GO:0001503~ossification | 4 | 0.0249 |
| GO:0033273~response to vitamin | 5 | 0.0037 | GO:0060348~bone development | 4 | 0.0323 |
| GO:0010033~response to organic substance | 13 | 0.0051 | **10. Regulation of blood vessel size (1.50)** |  |  |
| GO:0031667~response to nutrient levels | 6 | 0.0208 | GO:0035150~regulation of tube size | 3 | 0.0307 |
| GO:0048545~response to steroid hormone stimulus | 6 | 0.0235 | GO:0050880~regulation of blood vessel size | 3 | 0.0307 |
| GO:0009725~response to hormone stimulus | 8 | 0.0238 | GO:0003018~vascular process in circulatory system | 3 | 0.0331 |
| GO:0009991~response to extracellular stimulus | 6 | 0.0270 | **11. Sensory organ development (1.45)** |  |  |
| GO:0007584~response to nutrient | 5 | 0.0283 | GO:0048593~camera-type eye morphogenesis | 3 | 0.0252 |
| GO:0030155~regulation of cell adhesion | 4 | 0.0297 | GO:0007423~sensory organ development | 5 | 0.0491 |
| GO:0009611~response to wounding | 7 | 0.0313 | **12. Carboxylic acid transport (1.30)** |  |  |
| GO:0010647~positive regulation of cell communication | 6 | 0.0335 | GO:0046942~carboxylic acid transport | 4 | 0.0486 |
| Continue in the next column |  |  | GO:0015849~organic acid transport | 4 | 0.0494 |

Results of the functional annotation clustering performed using the DAVID's platform. Below each functional cluster (gray boxes) the GO clustered term (left columns), the number of differentially expressed genes that were present in each GO term (G, middle columns) and the statistical p value of GO term enrichment are indicatedment.
