# Supplementary material for: Gene Expression Changes in the Injured Spinal Cord Following Transplantation of Mesenchymal Stem Cells or Olfactory Ensheathing Cells
Source: PLoS One. 2013 Oct 11;8(10):e76141. doi: 10.1371/journal.pone.0076141 (PMC3795752; doi:10.1371/journal.pone.0076141)
Supplement: Table S15 — Functional annotation cluster: MSC 7.7 UP. (DOC) [file pone.0076141.s017.doc]

| **Table S15. Functional annotation cluster: MSC 7.7 UP** | | | | | |
| --- | --- | --- | --- | --- | --- |
| **Functional annotation cluster (enriched score)** | **G** | **P Value** | **Functional annotation cluster (enriched score)** | **G** | **P Value** |
| **1. Immune response (4.01)** |  |  | GO:0030097~hemopoiesis | 5 | 0.0011 |
| GO:0006955~immune response | 16 | 1.51E-16 | GO:0048522~positive regulation of cellular process | 11 | 0.0013 |
| GO:0002376~immune system process | 18 | 6.72E-16 | GO:0048534~hemopoietic or lymphoid organ development | 5 | 0.0017 |
| GO:0006952~defense response | 13 | 4.08E-12 | GO:0050863~regulation of T cell activation | 4 | 0.0017 |
| GO:0006954~inflammatory response | 10 | 2.59E-10 | GO:0002673~regulation of acute inflammatory response | 3 | 0.0017 |
| GO:0006950~response to stress | 16 | 6.22E-09 | GO:0002520~immune system development | 5 | 0.0020 |
| GO:0050896~response to stimulus | 22 | 9.15E-08 | GO:0002521~leukocyte differentiation | 4 | 0.0020 |
| GO:0009611~response to wounding | 10 | 9.81E-08 | GO:0050777~negative regulation of immune response | 3 | 0.0021 |
| GO:0009605~response to external stimulus | 12 | 2.35E-07 | GO:0050729~positive regulation of inflammatory response | 3 | 0.0021 |
| GO:0002682~regulation of immune system process | 9 | 5.84E-07 | GO:0051239~regulation of multicellular organismal process | 8 | 0.0034 |
| GO:0042110~T cell activation | 6 | 2.86E-06 | GO:0051249~regulation of lymphocyte activation | 4 | 0.0034 |
| GO:0045321~leukocyte activation | 7 | 3.60E-06 | GO:0046651~lymphocyte proliferation | 3 | 0.0034 |
| GO:0002252~immune effector process | 6 | 4.07E-06 | GO:0032943~mononuclear cell proliferation | 3 | 0.0036 |
| GO:0050776~regulation of immune response | 7 | 4.25E-06 | GO:0070661~leukocyte proliferation | 3 | 0.0036 |
| GO:0002684~positive regulation of immune system process | 7 | 6.01E-06 | GO:0032101~regulation of response to external stimulus | 4 | 0.0044 |
| GO:0031347~regulation of defense response | 6 | 7.39E-06 | GO:0002822~regulation of adaptive immune response based on somatic recombination of immune receptors built from immunoglobulin superfamily domains | 3 | 0.0075 |
| GO:0001775~cell activation | 7 | 7.74E-06 | GO:0002819~regulation of adaptive immune response | 3 | 0.0075 |
| GO:0002703~regulation of leukocyte mediated immunity | 5 | 1.46E-05 | GO:0031349~positive regulation of defense response | 3 | 0.0080 |
| GO:0046649~lymphocyte activation | 6 | 2.08E-05 | GO:0032103~positive regulation of response to external stimulus | 3 | 0.0085 |
| GO:0048583~regulation of response to stimulus | 8 | 2.33E-05 | GO:0002443~leukocyte mediated immunity | 3 | 0.0109 |
| GO:0002696~positive regulation of leukocyte activation | 5 | 0.0001 | GO:0048584~positive regulation of response to stimulus | 4 | 0.0114 |
| GO:0002697~regulation of immune effector process | 5 | 0.0001 | GO:0002526~acute inflammatory response | 3 | 0.0138 |
| GO:0050867~positive regulation of cell activation | 5 | 0.0001 | GO:0048513~organ development | 9 | 0.0144 |
| GO:0080134~regulation of response to stress | 6 | 0.0001 | GO:0008283~cell proliferation | 4 | 0.0154 |
| GO:0048518~positive regulation of biological process | 13 | 0.0002 | GO:0051707~response to other organism | 4 | 0.0162 |
| GO:0030217~T cell differentiation | 4 | 0.0003 | GO:0050789~regulation of biological process | 17 | 0.0163 |
| GO:0002706~regulation of lymphocyte mediated immunity | 4 | 0.0003 | GO:0048585~negative regulation of response to stimulus | 3 | 0.0193 |
| GO:0002694~regulation of leukocyte activation | 5 | 0.0003 | GO:0009607~response to biotic stimulus | 4 | 0.0269 |
| GO:0002704~negative regulation of leukocyte mediated immunity | 3 | 0.0004 | GO:0065007~biological regulation | 17 | 0.0359 |
| GO:0002707~negative regulation of lymphocyte mediated immunity | 3 | 0.0004 | GO:0007165~signal transduction | 8 | 0.0398 |
| GO:0050865~regulation of cell activation | 5 | 0.0004 | **2. Leukocyte adhesion (2.29)** |  |  |
| GO:0050870~positive regulation of T cell activation | 4 | 0.0005 | GO:0007159~leukocyte adhesion | 3 | 0.0011 |
| GO:0050727~regulation of inflammatory response | 4 | 0.0006 | GO:0022610~biological adhesion | 5 | 0.0111 |
| GO:0002683~negative regulation of immune system process | 4 | 0.0007 | GO:0007155~cell adhesion | 5 | 0.0111 |
| GO:0030098~lymphocyte differentiation | 4 | 0.0009 | **3. Response to steroid stimulus (1.50)** |  |  |
| GO:0002698~negative regulation of immune effector process | 3 | 0.0011 | GO:0048545~response to steroid hormone stimulus | 4 | 0.0216 |
| GO:0051251~positive regulation of lymphocyte activation | 4 | 0.0011 | GO:0043627~response to estrogen stimulus | 3 | 0.0364 |
| Continue in the next column |  |  | GO:0010033~response to organic substance | 6 | 0.0402 |

Results of the functional annotation clustering performed using the DAVID's platform. Below each functional cluster (gray boxes) the GO clustered term (left columns), the number of differentially expressed genes that were present in each GO term (G, middle columns) and the statistical p value of GO term enrichment are indicated.
