# Supplementary material for: Gene Expression Changes in the Injured Spinal Cord Following Transplantation of Mesenchymal Stem Cells or Olfactory Ensheathing Cells
Source: PLoS One. 2013 Oct 11;8(10):e76141. doi: 10.1371/journal.pone.0076141 (PMC3795752; doi:10.1371/journal.pone.0076141)
Supplement: Table S17 — Functional annotation cluster: MSC and OEC 7.7 UP. (DOC) [file pone.0076141.s019.doc]

| **Table S17. Functional annotation cluster: MSC and OEC 7.7 UP** | | | | | |
| --- | --- | --- | --- | --- | --- |
| **Functional annotation cluster (enriched score)** | **G** | **P Value** | **Functional annotation cluster (enriched score)** | **G** | **P Value** |
| **1. Immune response (9.39)** |  |  | GO:0002495~antigen processing and presentation of peptide antigen via MHC class II | 3 | 0.0002 |
| GO:0002376~immune system process | 14 | 5.88E-13 | GO:0019886~antigen processing and presentation of exogenous peptide antigen via MHC class II | 3 | 0.0002 |
| GO:0006955~immune response | 12 | 1.45E-12 | GO:0002504~antigen processing and presentation of peptide or polysaccharide antigen via MHC class II | 3 | 0.0002 |
| GO:0050896~response to stimulus | 15 | 7.46E-05 | GO:0002478~antigen processing and presentation of exogenous peptide antigen | 3 | 0.0004 |
| **2. Antigen processing and presentation (3.97)** |  |  | GO:0019884~antigen processing and presentation of exogenous antigen | 3 | 0.0006 |
| GO:0019882~antigen processing and presentation | 5 | 3.64E-06 | **3. Defense response (2.25)** |  |  |
| GO:0048002~antigen processing and presentation of peptide antigen | 4 | 1.97E-05 | GO:0006952~defense response | 5 | 0.0023 |
| Continue in the next column |  |  | GO:0002252~immune effector process | 3 | 0.0136 |

Results of the functional annotation clustering performed using the DAVID's platform. Below each functional cluster (gray boxes) the GO clustered term (left columns), the number of differentially expressed genes that were present in each GO term (G, middle columns) and the statistical p value of GO term enrichment are indicated.
