# Supplementary material for: Gene Expression Changes in the Injured Spinal Cord Following Transplantation of Mesenchymal Stem Cells or Olfactory Ensheathing Cells
Source: PLoS One. 2013 Oct 11;8(10):e76141. doi: 10.1371/journal.pone.0076141 (PMC3795752; doi:10.1371/journal.pone.0076141)
Supplement: Table S18 — Functional annotation cluster: MSC 7.7 DOWN. (DOC) [file pone.0076141.s020.doc]

| **Table S18. Functional annotation cluster: MSC 7.7 DOWN** | | | | | |
| --- | --- | --- | --- | --- | --- |
| **Functional annotation cluster (enriched score)** | **G** | **P Value** | **Functional annotation cluster (enriched score)** | **G** | **P Value** |
| **1. Lipid storage (3.72)** |  |  | **5. Regulation of cell differentiation (1.87)** |  |  |
| GO:0010886~positive regulation of cholesterol storage | 4 | 2.86E-06 | GO:0045597~positive regulation of cell differentiation | 8 | 0.0013 |
| GO:0010884~positive regulation of lipid storage | 4 | 9.92E-06 | GO:0051094~positive regulation of developmental process | 8 | 0.0043 |
| GO:0010744~positive regulation of foam cell differentiation | 4 | 1.58E-05 | GO:0022603~regulation of anatomical structure morphogenesis | 7 | 0.0046 |
| GO:0010885~regulation of cholesterol storage | 4 | 2.36E-05 | GO:0050793~regulation of developmental process | 12 | 0.0052 |
| GO:0010743~regulation of foam cell differentiation | 4 | 1.24E-04 | GO:0060688~regulation of morphogenesis of a branching structure | 3 | 0.0111 |
| GO:0010883~regulation of lipid storage | 4 | 0.0002 | GO:0016525~negative regulation of angiogenesis | 3 | 0.0111 |
| GO:0034381~lipoprotein particle clearance | 3 | 0.0038 | GO:0048518~positive regulation of biological process | 21 | 0.0155 |
| GO:0030301~cholesterol transport | 3 | 0.0182 | GO:0032879~regulation of localization | 10 | 0.0203 |
| GO:0015918~sterol transport | 3 | 0.0193 | GO:0030334~regulation of cell migration | 5 | 0.0223 |
| **2. Cell adhesion (3.37)** |  |  | GO:0048522~positive regulation of cellular process | 19 | 0.0226 |
| GO:0022610~biological adhesion | 11 | 0.0004 | GO:0051270~regulation of cell motion | 5 | 0.0331 |
| GO:0007155~cell adhesion | 11 | 0.0004 | GO:0040012~regulation of locomotion | 5 | 0.0336 |
| **3. Response vitamin and nutrient (3.25)** |  |  | GO:0006955~immune response | 7 | 0.0477 |
| GO:0033273~response to vitamin | 8 | 7.45E-06 | GO:0045595~regulation of cell differentiation | 8 | 0.0479 |
| GO:0007584~response to nutrient | 9 | 5.50E-05 | **6. Iron ion transport (1.81)** |  |  |
| GO:0033189~response to vitamin A | 6 | 6.62E-05 | GO:0000041~transition metal ion transport | 4 | 0.0059 |
| GO:0031667~response to nutrient levels | 10 | 8.29E-05 | GO:0006826~iron ion transport | 3 | 0.0120 |
| GO:0009991~response to extracellular stimulus | 10 | 1.42E-04 | GO:0030001~metal ion transport | 8 | 0.0133 |
| GO:0009605~response to external stimulus | 16 | 0.0002 | GO:0006811~ion transport | 10 | 0.0249 |
| GO:0032355~response to estradiol stimulus | 6 | 0.0002 | GO:0006812~cation transport | 8 | 0.0351 |
| GO:0032526~response to retinoic acid | 5 | 0.0004 | **7. Organ development (1.52)** |  |  |
| GO:0009719~response to endogenous stimulus | 12 | 0.0012 | GO:0048731~system development | 22 | 0.0217 |
| GO:0009725~response to hormone stimulus | 11 | 0.0016 | GO:0007275~multicellular organismal development | 24 | 0.0237 |
| GO:0010033~response to organic substance | 15 | 0.0025 | GO:0048513~organ development | 18 | 0.0305 |
| GO:0043627~response to estrogen stimulus | 6 | 0.0025 | GO:0048856~anatomical structure development | 22 | 0.0381 |
| GO:0048545~response to steroid hormone stimulus | 8 | 0.0027 | GO:0032502~developmental process | 25 | 0.0397 |
| GO:0001655~urogenital system development | 5 | 0.0193 | **8. Embryonic development (1.48)** |  |  |
| GO:0001822~kidney development | 4 | 0.0427 | GO:0035295~tube development | 6 | 0.0219 |
| **4. Lipid metabolic process (2.77)** |  |  | GO:0043009~chordate embryonic development | 7 | 0.0353 |
| GO:0042445~hormone metabolic process | 6 | 0.0007 | GO:0009792~embryonic development ending in birth or egg hatching | 7 | 0.0374 |
| GO:0034754~cellular hormone metabolic process | 5 | 0.0008 | GO:0009790~embryonic development | 9 | 0.0414 |
| GO:0016042~lipid catabolic process | 6 | 0.0018 | **9. Bone development (1.39)** |  |  |
| GO:0006629~lipid metabolic process | 12 | 0.0033 | GO:0001503~ossification | 4 | 0.0349 |
| GO:0010817~regulation of hormone levels | 6 | 0.0037 | GO:0060348~bone development | 4 | 0.0455 |

Results of the functional annotation clustering performed using the DAVID's platform. Below each functional cluster (gray boxes) the GO clustered term (left columns), the number of differentially expressed genes that were present in each GO term (G, middle columns) and the statistical p value of GO term enrichment are indicated.
