# Supplementary material for: Gene Expression Changes in the Injured Spinal Cord Following Transplantation of Mesenchymal Stem Cells or Olfactory Ensheathing Cells
Source: PLoS One. 2013 Oct 11;8(10):e76141. doi: 10.1371/journal.pone.0076141 (PMC3795752; doi:10.1371/journal.pone.0076141)
Supplement: Table S19 — Functional annotation cluster: OEC 7.7 DOWN. (DOC) [file pone.0076141.s021.doc]

| **Table S19. Functional annotation cluster: OEC 7.7 DOWN** | | | | | |
| --- | --- | --- | --- | --- | --- |
| **Functional annotation cluster (enriched score)** | **G** | **P Value** | **Functional annotation cluster (enriched score)** | **G** | **P Value** |
| **1. Extracellular matrix organization (2.76)** |  |  | GO:0007275~multicellular organismal development | 13 | 0.0029 |
| GO:0030198~extracellular matrix organization | 4 | 8.71E-04 | GO:0009653~anatomical structure morphogenesis | 8 | 0.0088 |
| GO:0043062~extracellular structure organization | 4 | 0.0033 | GO:0001568~blood vessel development | 4 | 0.0141 |
| **2. Positive regulation of cell adhesion (2.20)** |  |  | GO:0001944~vasculature development | 4 | 0.0153 |
| GO:0010811~positive regulation of cell-substrate adhesion | 3 | 0.0018 | GO:0048856~anatomical structure development | 11 | 0.0168 |
| GO:0010810~regulation of cell-substrate adhesion | 3 | 0.0038 | GO:0048513~organ development | 9 | 0.0231 |
| GO:0045785~positive regulation of cell adhesion | 3 | 0.0082 | GO:0048731~system development | 10 | 0.0326 |
| GO:0030155~regulation of cell adhesion | 3 | 0.0280 | **4. Regulation of cell growth (1.84)** |  |  |
| **3. Blood vessel development (1.97)** |  |  | GO:0001558~regulation of cell growth | 4 | 0.0067 |
| GO:0032502~developmental process | 14 | 0.0021 | GO:0040008~regulation of growth | 4 | 0.0306 |
| Continue in the next column |  |  |  |  |  |

Results of the functional annotation clustering performed using the DAVID's platform. Below each functional cluster (gray boxes) the GO clustered term (left columns), the number of differentially expressed genes that were present in each GO term (G, middle columns) and the statistical p value of GO term enrichment are indicated.
