# Supplementary material for: Gene Expression Changes in the Injured Spinal Cord Following Transplantation of Mesenchymal Stem Cells or Olfactory Ensheathing Cells
Source: PLoS One. 2013 Oct 11;8(10):e76141. doi: 10.1371/journal.pone.0076141 (PMC3795752; doi:10.1371/journal.pone.0076141)
Supplement: Table S20 — Functional annotation cluster: MSC and OEC 7.7 DOWN. (DOC) [file pone.0076141.s022.doc]

| **Table S20. Functional annotation cluster: MSC and OEC 7.7 DOWN** | | |
| --- | --- | --- |
| **Functional annotation cluster (enriched score)** | **G** | **P Value** |
| **1. Response to wounding (2.14)** |  |  |
| GO:0009605~response to external stimulus | 5 | 0.0066 |
| GO:0009611~response to wounding | 4 | 0.0076 |

Results of the functional annotation clustering performed using the DAVID's platform. Below each functional cluster (gray boxes) the GO clustered term (left columns), the number of differentially expressed genes that were present in each GO term (G, middle columns) and the statistical p value of GO term enrichment are indicated.
